# Nerve pathology and neuropathic pain after whiplash injury: a systematic review and meta-analysis

**DOI:** 10.1097/j.pain.0000000000002509

**Published:** 2021-10-12

**Authors:** Joel Fundaun, Melissa Kolski, Georgios Baskozos, Andrew Dilley, Michele Sterling, Annina B Schmid

**Affiliations:** 1 Nuffield Department of Clinical Neurosciences, The University of Oxford, Oxford, UK; 2 Department of Physical Therapy and Human Movement Sciences, Northwestern University, Feinberg School of Medicine, Chicago, IL, USA; 3 Shirley Ryan AbilityLab, Chicago, IL, USA; 4 Brighton and Sussex Medical School, University of Sussex, Brighton BN1 9PS, UK; 5 RECOVER Injury Research Centre, NHMRC Centre of Research Excellence in Recovery Following Road Traffic Injuries, The University of Queensland, Brisbane, Queensland, Australia

**Keywords:** Motor vehicle collision, whiplash associated disorder, traumatic neck pain, neuropathic pain, neuropathy

## Abstract

There is no clear understanding of the mechanisms causing persistent pain in patients with whiplash associated disorder (WAD). The aim of this systematic review was to assess the evidence for nerve pathology and neuropathic pain in patients with WAD. EMBASE, PubMed, CINAHL (EBSCO), and MEDLINE were searched from inception to 1^st^ September 2020. Study quality and risk of bias were assessed using the Newcastle-Ottawa Quality Assessment Scales. Fifty-four studies reporting on 390,644 patients and 918 controls were included. Clinical questionnaires suggested symptoms of predominant neuropathic characteristic in 34% of patients (range 25-75%). Mean prevalence of nerve pathology detected with neurological examination was 13% (0-100%) and 32% (10-100%) with electrodiagnostic testing. Patients independent of WAD severity (Quebec Task Force grades I-IV) demonstrated significantly impaired sensory detection thresholds of the index finger compared to controls, including mechanical (SMD 0.65 [0.30;1.00] p< 0.005), current (SMD 0.82 [0.25;1.39] p=0.0165), cold (SMD -0.43 [-0.73;-0.13] p=0.0204) and warm detection (SMD 0.84 [0.25;1.42] p=0.0200). Patients with WAD had significantly heightened nerve mechanosensitivity compared to controls upon median nerve pressure pain thresholds (SMD - 1.10 [-1.50;-0.70], p<0.0001) and neurodynamic tests (SMD 1.68 [0.92;2.44], p=0.0004). Similar sensory dysfunction and nerve mechanosensitivity was seen in WAD grade II, which contradicts its traditional definition of absent nerve involvement. Our findings strongly suggest a subset of patients with WAD demonstrate signs of peripheral nerve pathology and neuropathic pain. Although there was heterogeneity among some studies, typical WAD classifications may need to be reconsidered and include detailed clinical assessments for nerve integrity.

## Introduction

Whiplash associated disorders (WAD) commonly occur after motor vehicle crashes and often include signs and symptoms of pain, psychological distress, and sensory/motor dysfunction [97]. Currently, there is not a clear understanding of the mechanisms of persistent pain that occurs in approximately 50% of patients with WAD. Additionally, routine clinical testing and imaging do not typically identify a specific structural lesion causing pain or symptoms [24]. These clinical challenges are reflected by the overall small effects of current treatment strategies for these patients [122].

WAD is commonly classified using the Quebec Task Force severity grading scale [71] that grades severity from O (no pain and physical signs of injury) to IV (neck fracture/dislocation). The most common type is WAD grade II [49; 95], which includes neck symptoms and musculoskeletal signs (e.g., tenderness and impaired neck movement) in the absence of a frank nerve injury on routine diagnostic testing (electrodiagnostic tests, traditional neurological examination). However, individual WAD grades can include a diverse range of clinical signs and symptoms [16; 49; 95].

There is increasing evidence of nerve involvement and neuropathic pain in patients with chronic WAD. This includes sensory hypoaesthesia [17; 18], signs of nerve inflammation on magnetic resonance imaging (MRI) [44], and structural degeneration of small nerve fibres in skin biopsies [32]. Additionally, clinical questionnaires have identified some patients reporting neuropathic pain characteristics after whiplash injury [89; 106]. In line with these findings, a recent feasibility trial using a first-line neuropathic pain medication (pregabalin) for patients after acute whiplash injury showed short-term improvements in neck pain intensity when compared to placebo [70].

The presence of nerve pathology would have important implications for the management of patients with WAD. Compared to other chronic pain conditions, people with neuropathic pain experience greater impairments to quality of life and emotional wellbeing [3; 37]. Neuropathic pain and nerve pathology would also require targeted treatment approaches (e.g., neuropathic pain medication, specific physiotherapy methods) compared to non-neuropathic pain conditions [4]. Although there is emerging evidence, the involvement of nerve injury and neuropathic pain in WAD is not well understood. Thus, this systematic review aimed to assess whether there are indications of nerve pathology and neuropathic pain in patients after a whiplash injury.

## Methods

This review was preregistered on Prospero CRD42020211255; https://www.crd.york.ac.uk/prospero/display_record.php?ID=CRD42020211255) and was reported following the updated guidance for the Preferred Reporting Items for Systematic reviews and Meta-Analyses (PRISMA 2020) statement [74].

### 1 Eligibility

This review included observational studies (cross-sectional, cohort, and case-control) including measures of neuropathic pain and/or peripheral nerve pathology following motor vehicle crashes resulting in whiplash injuries. Studies were included if they reported on both 1) participants with WAD of any severity grade or duration; and 2) participants in whom measures of peripheral nerve pathology or neuropathic pain were reported. These could include a. Electrodiagnostic testing (e.g., nerve conduction, electromyography studies); b. Clinical examination findings of nerve pathology indicating loss of function (e.g., bedside neurological examination including muscle testing, sensory testing, reflexes); c. Quantitative sensory testing (specifically sensory measures of loss of function: mechanical, thermal, electrical detection); d. sympathetic reflexes (e.g., sympathetic skin responses); e. tests evaluating nerve mechanosensitivity (e.g., neurodynamic tests, pressure pain thresholds over peripheral nerves); f. imaging of neural structures (e.g., MRI, ultrasound); g. clinical questionnaires indicative of neuropathic pain (e.g., Self-complete Leeds Assessment of Neuropathic Symptoms and Signs (S-LANSS), Douleur Neuropathique 4 (DN4), Neuropathic Pain Symptom Inventory (NPSI)); h. grading systems or diagnostic codes suggesting the presence of nerve injury or neuropathic pain (e.g., NeuPSIG grading system, International Classification of Diseases (ICD) codes). Measures of peripheral nerve pathology or neuropathic pain had to be reported such that they could be either interpreted as stand-alone measures (e.g, bedside neurological testing, diagnostic codes), compared to a control group (e.g., QST) or previously published normative values (e.g., electrodiagnostic testing).

Exclusion criteria comprised studies not published in English, case series, conference abstracts and randomised controlled trials. Additionally, articles reporting on any of the following participant characteristics were excluded: 1) participants diagnosed with a central nervous system disorder or pathology (e.g., spinal cord injury, traumatic brain injury); 2) participants less than 18 years old; 3) participants with a previous diagnosis of peripheral neuropathy.

### 2 Search Strategy

EMBASE, PubMed, CINAHL (EBSCO), and MEDLINE were searched from inception to 1^st^ September 2020. A search strategy was developed by the study team in consultation with a medical librarian. The search strategies are provided in Supplemental Table S1 (available at http://links.lww.com/PAIN/B520).

### 3 Screening

Initial study eligibility was screened by one reviewer (JF) using titles/abstracts. Full texts were then reviewed by two independent reviewers (JF and MK). Disagreements in selection were resolved by consensus or consultation with a third reviewer (AS). Grey literature was searched for any additional articles by screening reference lists, theses (EThOS database), and policy documents. All studies were downloaded into EndNote referencing software (Clarivate, US) and duplicates were removed.

### 4 Data extraction

Data were extracted into a standardised excel spreadsheet developed and piloted by the study team. Extracted data included study characteristics (author, year, study design), sample size (WAD and controls), type and chronicity of WAD, the instrument or tool used to identify neuropathic pain/nerve pathology, as well as the type of outcome measures of neuropathic pain/nerve pathology in patients and healthy controls.

When possible, mean values and standard deviations (SD) relating to measures of neuropathic pain and nerve pathology were extracted for patients and healthy controls. Extracted data lacking a control group was compared to published normative values (e.g, questionnaire cut-off scores, electrodiagnostic testing) or to referenced diagnostic criteria (e.g., ICD codes). Where included information was unclear, we attempted to contact the authors to obtain the necessary information. If studies reported alternative summary statistics, means and SD were transformed using recommended calculations [117]. Graphically reported means and SD were estimated using Plot Digitizer software [54], as recommended by the Cochrane Handbook [52]. Data were extracted by one reviewer (JF) and independently checked by another reviewer (MK).

We further categorised studies (not individual patients) according to the Neuropathic Pain Grading System published by the Neuropathic Pain Special Interest Group of the International Association for the Study of Pain [37] to gather information about the certainty of neuropathic pain. Data extraction included details regarding each criterion on the grading system. Possible neuropathic pain included a history suggesting relevant neurologic lesion and a neuroanatomically plausible pain distribution. We assumed the history of a whiplash injury itself has the potential to include nerve involvement for a subset of patients [71] and that pain referral to the neck or upper limbs is neuroanatomically plausible as the forces acting on the neck could affect neural structures multisegmentally [12; 24]. Probable neuropathic pain included negative sensory signs in the same neuroanatomically plausible distribution, such as identified with quantitative sensory testing or bedside neurological examination. Definite neuropathic pain included a diagnostic test confirming a lesion or disease of the somatosensory nervous system explaining the pain, such as electrodiagnostic tests and imaging of neural structures. A grading of the next higher category could only be reached if the previous categories were met. If diagnostic tests confirmed a nerve lesion on diagnostic tests (e.g., MRI) but sensory signs were not assessed, we classed these studies into a separate category of ‘nerve pathology’.

### 5 Quality assessment

Study quality and risk of bias were assessed using the Newcastle-Ottawa Quality Assessment Scales (NOS) for case-control and longitudinal cohort studies. These are scored from zero to nine for the categories of study selection, comparability, and exposure or outcome. For cross-sectional studies, an adapted NOS [120] was used, which is scored out of 10. The NOS classifies the risk of bias of observational studies on an increasing scale, with higher scores reflecting a lower risk of bias. Whereas no recommended cut-offs exist for case-control and cohort studies, NOS cross-sectional studies were interpreted using a previously described method [120] with scores from 0–3 indicating high risk, 4–7 as moderate risk, and 8–10 as low risk. Two independent reviewers assessed each study for risk of bias (JF and MK). Disagreements between reviewers were resolved through consensus or by mediation of a third reviewer (AS).

### 6 Data synthesis and analysis

Results not included in the meta-analyses are described using narrative synthesis of nerve pathology or neuropathic pain measures. We used the Guidance on the Conduct of Narrative Synthesis in Systematic Reviews: A Product from the ESRC Methods Programme (2006) to report our findings [77].

If data were available for the same outcome measure from at least 2 studies using similar assessment methodology, meta-analysis was performed. Two meta-analyses were performed: 1) summarising overall data from all studies independent of WAD grade and 2) summarising studies only including patients with WAD I-II who per definition should not demonstrate nerve pathology [71]. If outcome measures from at least two studies examined more than one anatomical site (e.g., detection thresholds at finger and neck), each site was meta-analysed separately. If studies reported outcome measures for both right and left sides in the same participants, pooled means and SD were reported to avoid inflation during meta-analysis.

All statistical calculations were performed using the freely available software R [113] and RStudio [114] using the packages ‘Meta’ and ‘Metafor’ [47]. For estimated prevalence data, means and ranges were reported. For continuous data, group means, SD, and sample sizes were used to calculate standardised mean differences (SMD) and 95% confidence intervals (CI). P-values and I^2^ heterogeneity were also reported.

Random effects models and inverse variance weighting methods were used to account for the variability of included studies. Statistical significance between patients and healthy control participants was determined using t-tests with a pre-registered significance cut-off of p-value < 0.05. The Hartung-Knapp adjustment for random effects model and Hedges’ g bias correction for standardised mean difference were used. Sidik-Johnkman estimator for tau^2^ adjusted for between study variance. As a very small number of studies can make it impossible to estimate the between-studies variance with precision, a fixed effects model was used if only 2 studies were meta-analysed [9]. Heterogeneity was calculated using I^2^ statistics and interpreted as ‘might not be important’ (0-40%), ‘moderate’ (30-60%), ‘substantial ‘(50-90%), and ‘considerable’ (75-100%) [52].

## Results

**Figure 1:**
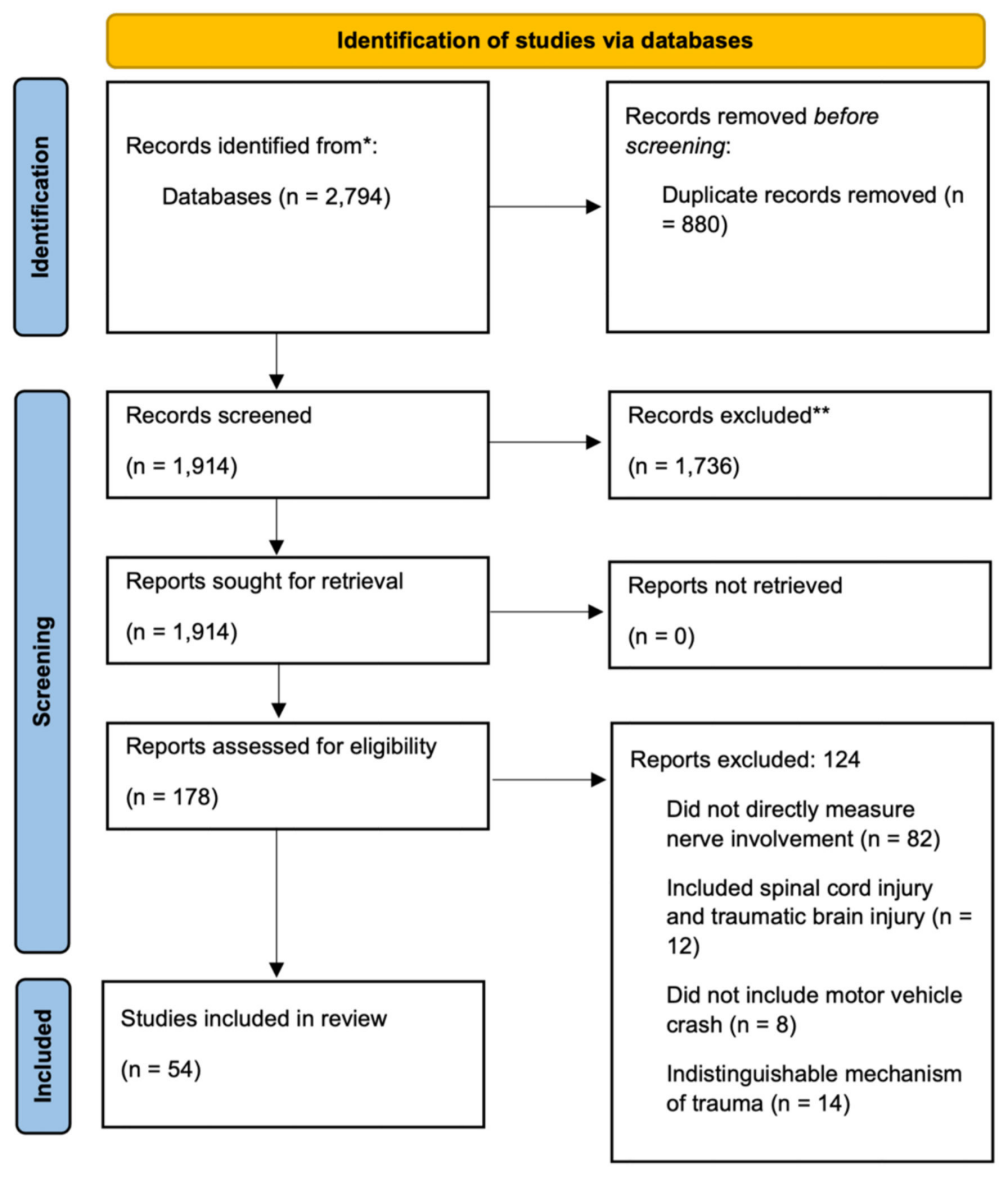
PRISMA Flow Diagram

The search identified 1,914 non-duplicate citations for abstract/titles screening. A total of 178 articles were screened for full-text eligibility. A total of 54 studies reporting on n=390,644 patients and n=918 controls were included in this review (Figure 1). The main reason for study exclusion was the absence of a direct measure of nerve pathology or neuropathic pain (82 studies). We attempted to contact the authors of two studies for details regarding inclusion criteria and study methodology [85; 116]. As we did not receive any responses, these studies were not included in this review.

Detailed study characteristics can be seen in Table 1 and Supplemental Table S2 (available at http://links.lww.com/PAIN/B520). The studies included a range of observational designs (22 cross-sectional, 28 cohort, four case-control), and reported on sample sizes between n=9 and n=384,539 patients/controls. The average age of WAD participants was 37.67 (SD 2.25) years and 42.7% were female.

Thirty-two of the 54 included studies (59%) reported the grade of WAD severity using the Quebec Task Force grading scale (0-4) [71]. The most commonly reported was WAD grade II (7 studies, n=307 total patients) followed by the combination of grades II-III (6 studies, n=408 total patients) and grades I-III (5 studies, n=283 total patients).

Sensory detection measures were identified for six major body sites. We grouped outcomes recorded over the thenar eminence, phalange I and metacarpophalangeal joint I into a meta-analysis for ‘thumb’; the phalanges II and metacarpophalangeal joint II into a meta-analysis for ‘index finger’; and the phalanges V and hypothenar muscle into a meta-analysis for ‘little finger’. Two studies [90; 121] reported outcome measures using separated values for right and left sides, which were pooled to avoid inflation during meta-analysis.

## Quality assessment

NOS is summarised in Supplemental Table S3 (available at http://links.lww.com/PAIN/B520). The median score was 7 (range 3-10) for cross-sectional studies, 5 (range 3-8) for cohort studies, and 5.5 (range 5-6) for case-control studies indicating a moderate risk of bias on average, with studies ranging from low to high risk of bias. The comparability of subjects and controls based on study design was the most common limitation. The total score agreement between raters was 87.7%.

## Evidence of nerve pathology and neuropathic pain in WAD I-IV

In total, 19 assessments were utilised to assess neuropathic pain or peripheral nerve pathology. The use of normative values was not required as all meta-analysed studies included their own control groups.

**Figure 2:**
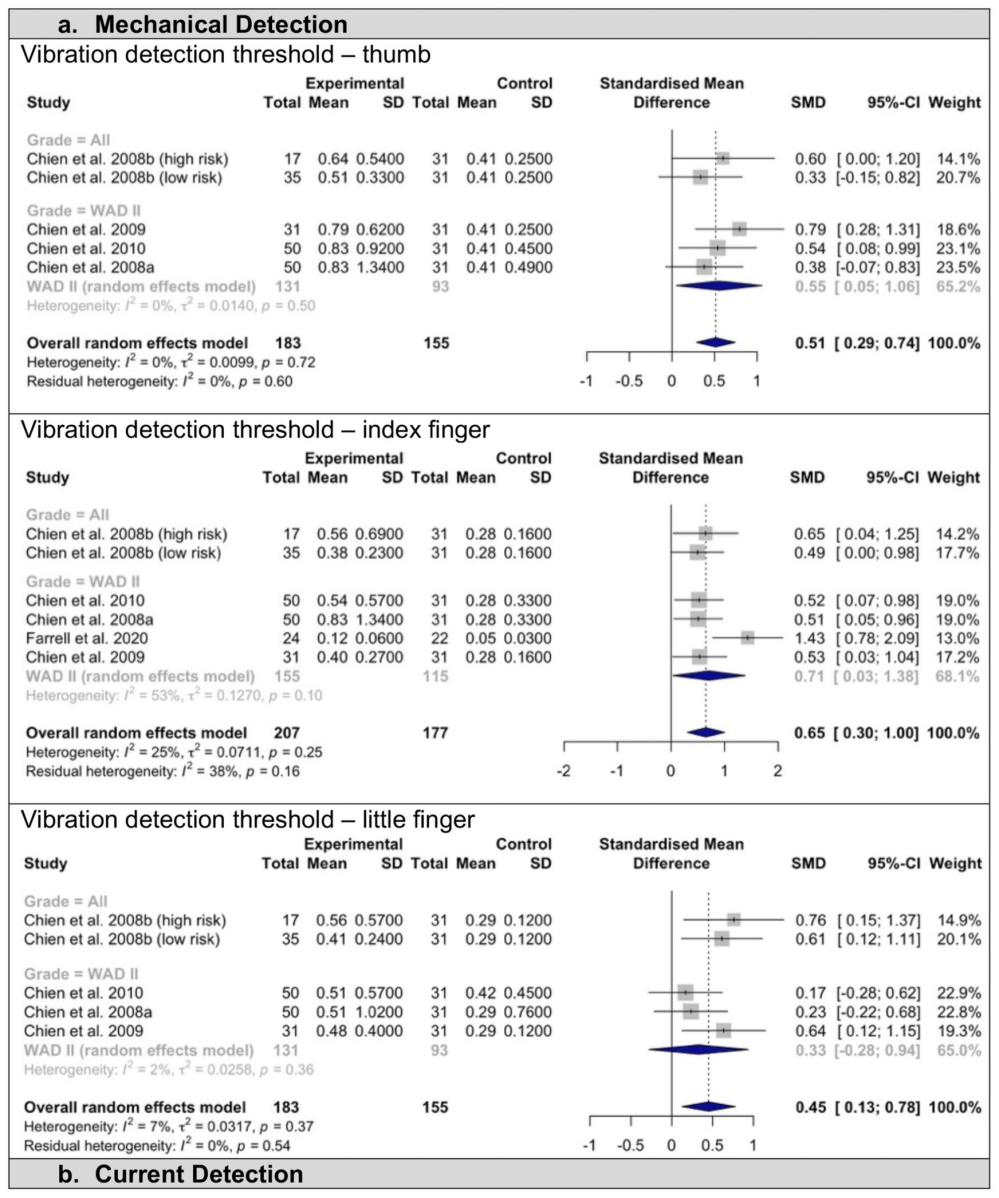

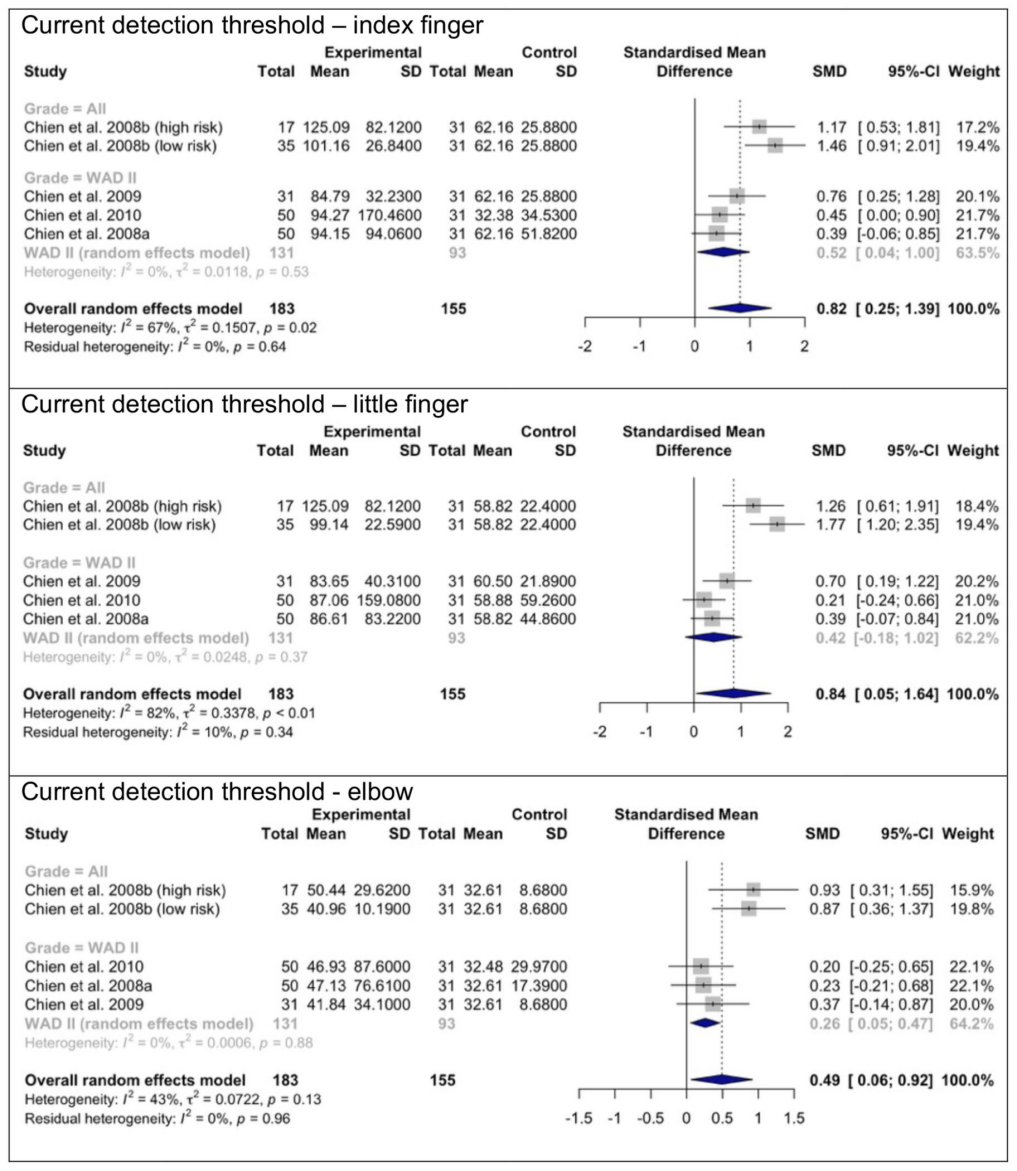

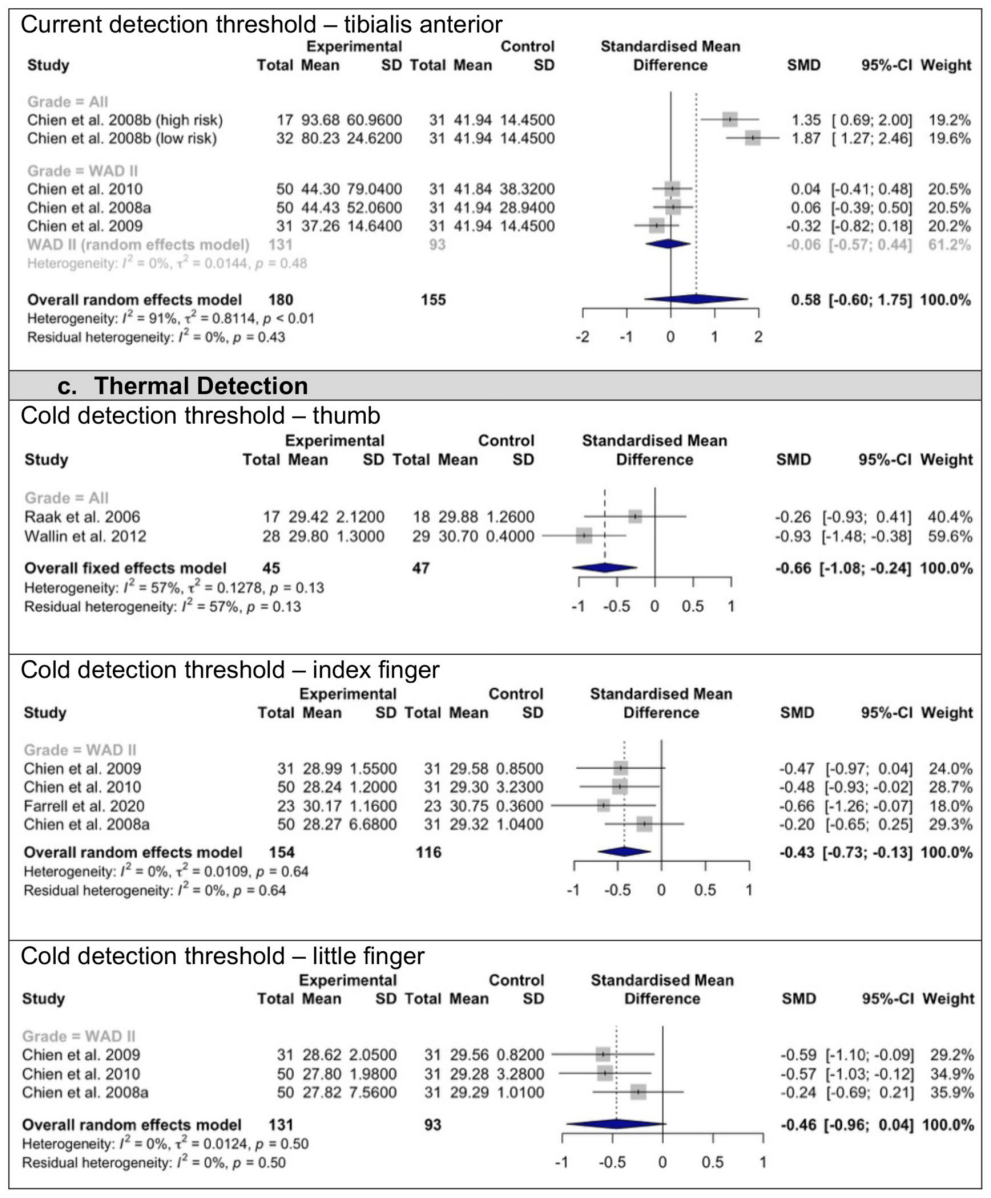

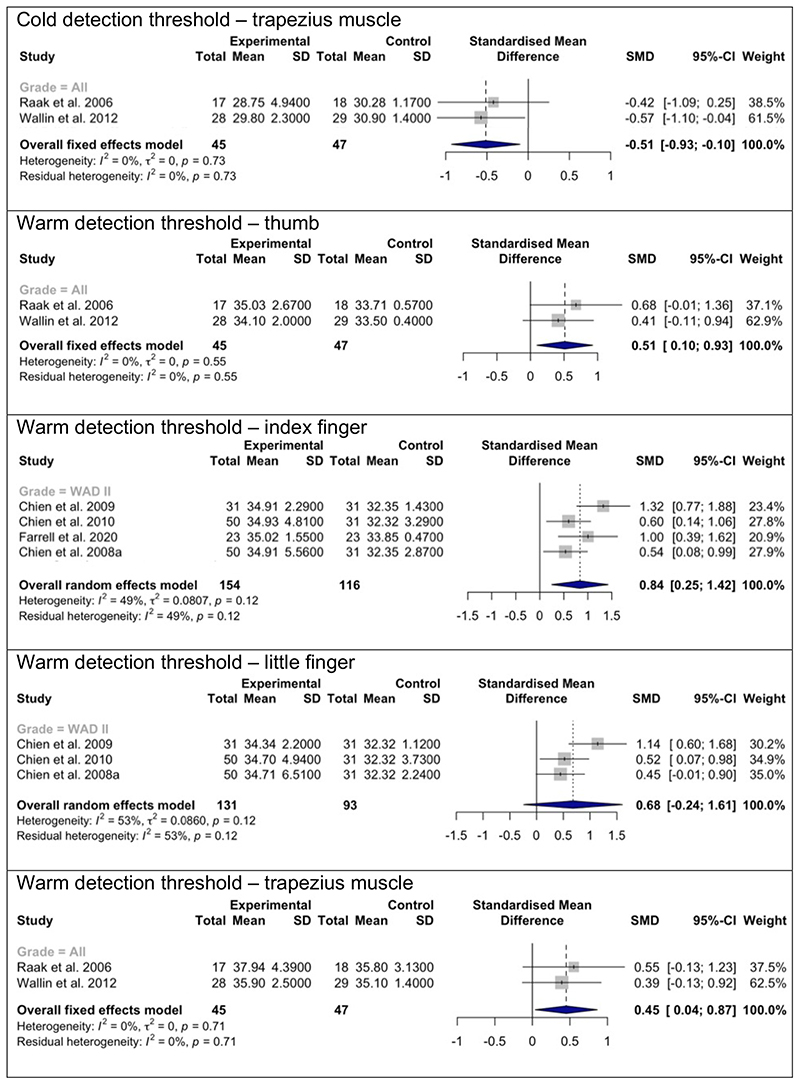

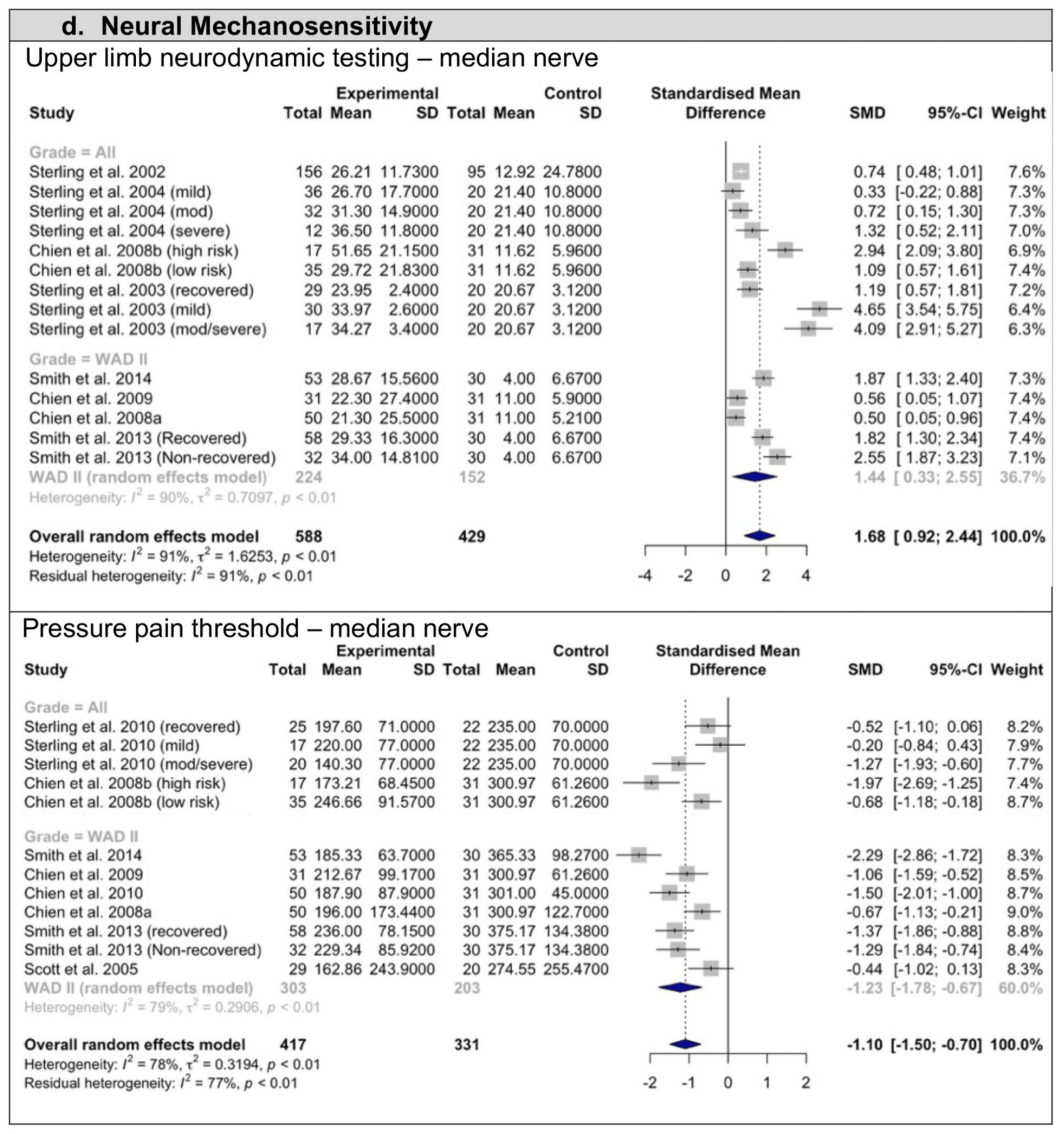
Meta-analysis of detection threshold measures and neural mechanosensitivity. Studies are subgrouped based on the Quebec Task Force grading scale. Overall effects, standardised mean differences (SMD), 95% confidence intervals (CI), and I^2^ heterogeneity are summarised for two meta-analyses: 1) including the overall data from all studies independent of WAD grades (“All”) and 2) for studies only including patients with grade II (“WAD II”).

The findings of studies including all WAD severity grades (I-IV) are categorised by type of outcome measure (Figure 2 and Supplemental Table S4, available at http://links.lww.com/PAIN/B520). Mechanical, current, and thermal detection thresholds were measured at multiple sites including the thumb, index finger, little finger, upper trapezius muscle, and anterior tibialis muscle and were meta-analysed separately. Neural mechanosensitivity of the median nerve included data on upper limb neurodynamic testing (measured as degrees of elbow flexion) and pressure pain thresholds measured over peripheral nerves (PPT; using an algometer). Individual studies that reported participant subcategories (e.g., mild pain vs moderate/severe pain, recovered vs non-recovered, etc) were indicated in the analyses.

The most commonly used assessments for nerve pathology after whiplash injury were PPT over peripheral nerves and nerve palpation (17 studies, [2; 15–18; 44; 45; 75; 88; 90; 91; 96; 101–105]), electrodiagnostic testing (16 studies, [2; 11; 12; 19; 20; 22; 50; 56; 57; 62; 67; 68; 73; 83; 94; 115]), and clinical neurological examination (16 studies, [2; 32; 44; 55; 58; 62; 66; 73; 76; 79; 80; 92; 107–110]. Four studies [101; 102; 104; 105] assessed sympathetic vasoconstrictor responses. Two studies used diagnostic ICD-9 coding for nerve injury and involvement [7; 83]. Additional assessments of nerve pathology from single studies included cutaneous silent periods [62], laser evoked potentials [43], intraepidermal nerve fibre density [32], MRI [44], and ultrasound [45] (Table 1 and Supplemental Table S4, available at http://links.lww.com/PAIN/B520).

### Prevalence of neuropathic pain

The prevalence of neuropathic pain signs and symptoms was determined in five studies by two questionnaires (S-LANSS and DN4). The prevalence scores indicating the presence of neuropathic pain characteristics had a mean of 34% (range 25-75%, n=208 in all grades of WAD severity [32; 44; 89; 90; 106]. Two studies used the NPSI to evaluate the severity of neuropathic pain symptoms with a median score of 3 out of 10 (interquartile range: 6, n=20) [89] and mean score of 26.1 out of 100 (SD 18.3, n=24) [32]. See Table 1 and Supplemental Table S4 for a summary of study assessments and outcomes (available at http://links.lww.com/PAIN/B520).

Table 2 includes a summary of the certainty of neuropathic pain for each study according to the neuropathic pain grading system. Five studies (9.3%) included sufficient tests so that a grading of definite neuropathic pain could be reached at least in a subgroup of patients. Nineteen studies (35.2%) could reach a grading of probable and 18 (33.3%) of possible neuropathic pain. Results from 12 studies (22.2%) were classed as ‘nerve pathology’ as the absence of sensory testing in the presence of a confirmatory diagnostic tests prevented a firm conclusion of definite neuropathic pain.

### Prevalence of nerve pathology

The mean prevalence of nerve pathology identified by clinical examination varied according to the assessment used: neurological examination was 13% (range 0-100%, n=1,885) [2; 32; 44; 55; 58; 62; 66; 73; 76; 79; 80; 92; 107–110]) and electrodiagnostic testing was 32% (range 10-100%, n=3,921) [2; 11; 12; 19; 20; 22; 50; 56; 57; 62; 67; 68; 73; 83; 94; 115]). ICD-9 codes related to nerve pathology and nerve injury included n=384,617 patients from two studies with a nerve injury mean prevalence of 1% (range 1-100%) [7; 83].

### Mechanical Detection

All three locations where vibration detection thresholds were reported demonstrated significantly impaired vibration thresholds in patients compared to controls (Figure 2a). This difference was significant at all locations measured in the hand, including the thumb (SMD 0.51 [0.29; 0.74] p=0.0032, I^2^ = 0%), index finger (SMD 0.65 [0.30; 1.00] p<0.005, I^2^ = 25%), and little finger (SMD 0.45 [0.13; 0.78] p=0.0183, I^2^ = 7%) compared to controls with heterogeneity that may not be considered important. One study showed a statistically significant decrease in mechanical detection thresholds using von Frey hairs but not mechanical pain threshold at the index finger compared to healthy controls (Table 1) [32].

### Current Detection

Studies measuring current detection thresholds found significant differences at the index finger (SMD 0.82 [0.25; 1.39] p=0.0165, I^2^ =67%), little finger (SMD 0.84 [0.05; 1.64] p=0.0425, I^2^ =82%), and elbow (SMD 0.49 [0.06; 0.92] p=0.0337, I^2^ = 43%). However, the current detection threshold over the tibialis anterior muscle was not statistically significant between patients and controls (SMD 0.58 [-0.60; 1.75] p=0.2435, I^2^ = 91%). All current detection measures had moderate to considerable between study heterogeneity (Figure 2b).

### Thermal Detection

In total, six studies measured thermal detection in multiple upper extremity locations (Figure 2c). Cold detection thresholds were significantly impaired at the thumb (SMD -0.66 [-1.08; -0.24] p=0.0023, I^2^=57%), index finger (SMD -0.43 [-0.73; -0.13] p=0.0204, I^2^ =0%), and trapezius muscle (SMD -0.51 [-0.93; -0.10] p= 0.0154, I^2^=0%), but not at the little finger (SMD -0.46 [-0.96; 0.04] p=0.0574, I^2^ = 0%) in patients compared to controls.

Warm detection thresholds showed significant impairments at the thumb (0.51 [0.10; 0.93] p=0.0161, I^2^=0%), index finger (SMD 0.84 [0.25; 1.42] p=0.0200, I^2^ = 49%), and trapezius muscle (SMD 0.45 [0.04; 0.87] p=0.0329, I^2^=0%), but not at the little finger (SMD 0.68 [-0.24; 1.61] p=0.0866, I^2^ = 53%). Between-study heterogeneity ranged from not considered important to moderate. Thermal detection thresholds at the tibialis anterior muscle were measured in one study [121], which found a significant impairment in left-sided but not right-sided warm detection compared to controls.

### Neural Mechanosensitivity

Eight studies and a total of n=527 patients and n=389 healthy controls were included in the neural mechanosensitivity meta-analysis. A significant difference is seen in both elbow range of motion during median nerve neurodynamic testing (SMD 1.68 [0.92; 2.44], p=0.0004, I^2^ = 91%) and PPT over the median nerve at the elbow (SMD -1.10 [-1.50; -0.70], p<0.0001, I^2^ =78%) compared to controls (Figure 2d); both with considerable between-study heterogeneity. The average proportion of patients who reported symptom reproduction upon median nerve palpation was 91% (range 67-100%, n=56 total patients) [2; 44; 45] and 94% (range 78-100%, n=50 total patients) upon brachial plexus palpation [44; 45; 65].

### Other assessments

Four studies (n=293) [101; 102; 104; 105] assessed sympathetic vasoconstrictor response with a mean quotient of integral of 59.42 (SD 7.13) and sympathetic reflex quotient of 0.72 (SD 0.70) listed in Supplemental Table S4 (available at http://links.lww.com/PAIN/B520). One study (n=20) assessing cutaneous silent periods found abnormalities suggestive of peripheral nerve involvement [62]. In contrast, another study (n=21) measuring laser evoked potentials did not find a difference between patients with WAD I-III and healthy controls [43]. Five additional studies used sensory testing parameters that were not comparable for meta-analysis [53; 69; 75; 108; 119] but most findings consistent with the presence of a sensory deficit; complete outcome details provided in Table 1.

Two imaging studies both reported signs of nerve involvement. Using MRI, one study found greater T2 weighted signal intensity of the brachial plexus and median nerve at the wrist compared to controls [44]. Another imaging study using high frequency ultrasound identified biomechanical changes to median nerve excursion at the forearm and wrist [45]. Lastly, a significant decrease in intraepidermal nerve fibre and dermal nerve bundle densities were apparent in skin biopsies of the index finger compared to controls [32].

## Evidence of nerve pathology and neuropathic pain in WAD II

Eight studies reported separate data for patients classified as only WAD grade II and were sub-grouped for meta-analysis (Figure 2 and Supplemental Table S4, available at http://links.lww.com/PAIN/B520). Additional assessments of peripheral nerve pathology in WAD II included mechanical detection using von Frey hairs [32]; T2 weighted signal intensity of the peripheral nerves using MRI [44]; biomechanical changes to nerve excursion using high frequency ultrasound [45]; and structural intraepidermal nerve fibre and dermal nerve bundle density using skin biopsies [32].

### Prevalence of Neuropathic Pain

Using the S-LANSS, mean prevalence scores indicating the presence of neuropathic pain characteristics were 34% (range 25-36%, n=123) in WAD II [32; 44; 90]. One study used the NPSI and reported a mean (SD) of 26.1 (18.3) out of 100 (n=24) [32].

Using the IASP neuropathic pain grading system, two of the 8 studies (25%) had sufficient tests to reach the grade of definite neuropathic pain in at least a subgroup of patients. Results from three studies (38%) reached a grade of probable neuropathic pain and another three studies (38%) could reach a grade of possible neuropathic pain. As all studies included reports of pain and sensory testing, no studies were classed as ‘nerve pathology’ (Table 2).

### Mechanical Detection

Vibration detection thresholds were measured at the thumb, index and little fingers (Figure 2a). Overall, there were significantly impaired vibration detection thresholds at the thumb (SMD 0.55 [0.05; 1.06] p=0.0422, I^2^ = 0%) and index finger (SMD 0.71 [0.03; 1.38] p= 0.0446, I^2^ = 53%), but no difference at the little finger (0.33 [-0.28; 0.94] p=0.1448, I^2^ =2%) compared to controls. Heterogeneity ranged from might not be important to moderate. As previously reported, one study including only WAD II found a significant reduction in mechanical detection using von Frey hairs but preserved mechanical pain at the index finger compared to controls [32].

### Current Detection

Current detection thresholds of WAD II were significantly higher at the index finger (SMD 0.52 [0.04; 1.00] p=0.0427, I^2^ = 0%) and elbow (SMD 0.26 [0.05; 0.47] p=0.0332; I^2^ = 0%), but not at the the little finger (SMD 0.42 [-0.18; 1.02] p=0.0961, I^2^ = 0%) or tibialis anterior muscle (SMD -0.06 [-0.57; 0.44] p=0.6537, I^2^ = 0%) compared to healthy controls (Figure 2b). Overall heterogeneity was very low.

### Thermal Detection

The previously described thermal detection thresholds for the index and little fingers included only WAD II and can be seen in Figure 2c.

### Neural Mechanosensitivity

Six studies reported PPT of the median nerve at the elbow and four studies reported median nerve neurodynamic testing (Figure 2d). Compared to controls, there was significantly restricted elbow range of motion during median nerve neurodynamic testing (SMD 1.44 [0.33; 2.55] p=0.0225, I^2^ = 90%) and lower median nerve PPT (SMD -1.23 [-1.78; -0.67] p=0.0016, I^2^ =79%) in patients with WAD II. Both analyses demonstrate substantial heterogeneity. The proportion of patients who reported symptom reproduction upon nerve palpation of the brachial plexus and median nerve ranged from 78-88.9% and 55.6-66.7%, respectively in two studies (n=18) [44; 45].

### Other assessments

Single studies using MRI, high frequency ultrasound and skin biopsies all found indications of nerve involvement (Table 1 and Supplemental Table S4, available at http://links.lww.com/PAIN/B520).

## Discussion

Our systematic review including 54 studies in 390,644 patients suggests that after whiplash injury, a subset of people demonstrate signs of peripheral nerve injury and/or neuropathic pain. These findings were seen irrespective of whiplash severity grading, and importantly, were also present in WAD II. These data contradict the traditional definition of WAD II, which is defined by an absence of nerve involvement. The included studies utilised a varied set of clinical measures and questionnaires to identify signs of nerve pathology and neuropathic pain. The mean prevalence estimates of nerve pathology in WAD ranged from 1% (ICD-9 codes) to 32% (electrodiagnostic testing). The prevalence of neuropathic pain determined with questionnaires ranged from 34% to 75%. Measures of nerve function revealed abnormalities in large nerve fibres apparent by the presence of muscle weakness, hyporeflexia, hypoaesthesia to light touch and vibration, and abnormal electrodiagnostic testing. Small nerve fibre pathology was recognised via reduced temperature, pin prick, current detection thresholds, and decreased intraepidermal nerve fibre density. Several studies demonstrated heightened nerve mechanosensitivity, and imaging studies suggested altered nerve movement and structural abnormalities using high frequency ultrasound and MRI, respectively.

## Neuropathic pain is reported by a significant group of patients with WAD

Pooled from four studies and 208 patients, the S-LANSS identified 34% of patients with predominant neuropathic pain characteristics. When using the DN4 questionnaire, one study found estimates of neuropathic pain as high as 75% in a smaller sample size (n=20) [89]. The prevalence of neuropathic pain appears in contrast to the low prevalence of nerve pathology from ICD-9 codes (1%). This disparity, though, is primarily based on one large retrospective study (n=384,539) using ICD-9 codes which only included peripheral nerve injuries in WAD that were present with an accompanying upper or lower extremity fracture [7]. Conversely, estimates of neuropathic pain from questionnaires closely align with clinical signs of nerve pathology identified during electrodiagnostic testing (32%).

The neuropathic pain grading system [37] helps to determine the certainty of neuropathic pain. Unfortunately, no study used the grading system at individual patient level. We therefore performed retrospective grading at study level, thus providing information about at least a subset of patients. Thirty-five percent of studies reached a grading of probable neuropathic pain by providing evidence of sensory signs in the upper extremity or neck predominantly through quantitative sensory testing which is considered as an examination to detect sensory signs in the grading system [37]. Although sensory signs and symptoms were reported from neuroanatomically plausible areas, retrospective analysis cannot conclusively confirm these findings were a result of direct nerve involvement. Intriguingly though, 31% of studies confirmed a lesion of the somatosensory nervous system through diagnostic tests (e.g., electrodiagnostic tests, MRI). As many of these studies (22%) did not include sensory testing, we took a conservative approach and only classified five (9.3%) as ‘definite’ neuropathic pain.

Taken together, the data from questionnaires and retrospective neuropathic pain grading at study level suggest that a significant portion of patients with WAD experience at least probable neuropathic pain. This illustrates the importance of clinical screening for neuropathic pain symptoms in this population.

## Sensory loss of function is apparent across a range of modalities

A hallmark of nerve pathology and peripheral neuropathic pain is the presence of sensory loss of function in the anatomical territory of the suspected lesion of the peripheral nervous system [37]. We did not include gain of function measures (thermal and mechanical pain thresholds, wind-up ratios, etc) as hyperalgesia is not only a feature of neuropathic but also nociceptive [14; 36] or nociplastic pain [8; 21]. Overall, the sensory testing results show a loss of function affecting both large (vibration, light touch) and small nerve fibres (temperature) in patients with WAD compared to healthy controls. Sensory dysfunction was present throughout the entire upper extremity, but most consistently seen in the thumb and index finger. Lower extremity sensory assessment included current and thermal detection thresholds at the tibialis anterior, which was not significantly different from controls. This suggests there is reduced sensory function in the upper extremity in at least a subset of patients after whiplash injury.

Similar findings of loss of function dominate a range of focal nerve injuries, including lumbar radiculopathy [112], carpal tunnel syndrome [6], and various traumatic peripheral nerve lesions [51]. As such, a direct nerve injury resulting from the collision may explain the identified loss of function. The theory that whiplash injury causes peripheral nerve injury in some patients is supported by sensory testing, neurological examination, and electrodiagnostic testing [15; 50; 80]. Both preclinical and clinical data suggest sensory hypoaesthesia [84] can occur as early as one week after peripheral nerve injury. These sensory abnormalities may indicate functional or structural nerve pathology, such as ischaemia [23; 111], demyelination or axon degeneration [46; 63]. In line with this hypothesis, a single study taking skin biopsies demonstrated structural nerve fibre loss in chronic WAD [32].

Alternatively, upper extremity sensory loss of function may be a downstream effect that develops from secondary mechanisms rather than from a direct nerve injury. Indeed, subtle sensory hypoaesthesia has been identified in non-neuropathic conditions [40; 64]. It has been speculated that such hypoaesthesia in the absence of an apparent nerve lesion could be attributed to central mechanisms [30], which are known to not only modulate painful but also non-painful sensory input [29; 64].

Another potential secondary mechanism that might explain sensory loss of function is inflammatory processes triggered after a motor vehicle crash [61; 99; 100]. Elevated systemic inflammation has previously been linked with widespread sensory hypoaesthesia in other painful conditions such as fibromyalgia [33] and complex regional pain syndrome [41]. Preclinical models of traumatic nerve injury suggest that pathological neuroinflammation has a role in inducing axonal degeneration [48; 59]. This hypothesis is supported by radiological findings of increased T2 signal intensity of the brachial plexus and median nerve in patients with chronic WAD [44], which has been interpreted as a clinical correlate of neuroinflammation [93]. Additionally, increased levels of serum inflammatory markers have been identified from patients with chronic WAD [99; 100].

As such, systemic or central mechanisms, in addition to direct traumatic nerve injury, may explain the reported sensory abnormalities in WAD. Further studies evaluating the temporal development and spatial distribution of neural loss of function could shed light on the nature of mechanisms driving the consistent sensory hypoaesthesia.

## Clinical findings of nerve mechanosensitivity are present in some patients after whiplash injury

This review identified the presence of heightened median nerve mechanosensitivity to nerve elongation or pressure. Such nerve mechanosensitivity in patients is consistent with findings of nociceptive axonal mechanical sensitivity reported in animal models of localised peripheral neuroinflammation [10; 26; 42]. Although these findings may demonstrate nerve involvement, they do not necessarily confirm direct nerve pathology or neuropathic pain as nerve mechanosensitivity can also be present in patients without apparent nerve injury. Consistent with this, PPT over peripheral nerves has shown heightened sensitivity in both neuropathic [16; 34; 35] and traditionally non-neuropathic pain conditions, such as tension-type headache [13] and epicondylalgia [35]. Furthermore, upper limb neurodynamic tests do not demonstrate diagnostic accuracy in detecting peripheral neuropathic pain [60] as they can be negative in patients with clear nerve involvement [5] or positive in patients with traditionally non-neuropathic conditions such as non-specific neck and arm pain [72] and fibromyalgia [118]. Therefore, although the findings of heightened nerve mechanosensitivity in WAD are intriguing and warrant further exploration, care must be taken in their interpretation regarding neuropathic pain or structural nerve pathology.

## Neuropathic pain and nerve dysfunction are present irrespective of WAD severity grading

Whereas the presence of nerve pathology and neuropathic pain may not be surprising in patients with WAD III (defined by the presence of neurological signs), our findings suggest there is nerve involvement even in some patients with WAD II. This was apparent by the self-reports of neuropathic pain in 34% of WAD II patients (LANSS) [32; 44; 90]. In addition, multiple measures showed abnormal findings, including reduced neural excursion on ultrasound [45] and increased T2 weighted signal intensity on MRI [44], reduced nerve fibre density from skin biopsy [45], and measures of sensory hypoaesthesia [16–18; 32]. Of note, the findings in the WAD II cohort were comparable to the analysis including all WAD grades, suggesting that the findings are not purely driven by more severe WAD grades.

Our findings directly challenge the widely used Quebec Task Force definition, in which patients with WAD II are characterised by musculoskeletal signs including decreased range of motion and point tenderness in the absence of neurological deficits [71]. The Quebec Task Force classification system has long received criticism regarding its over-simplified classifications [25; 38] with suggestions to modify grade II [49]. Alternative classifications have been proposed incorporating recent advances in psychological and physiological variables related to recovery [28; 95]. Nevertheless, the original Quebec Task Force grading system remains popular because of its simplicity [98]. This may be contributing to the diagnostic difficulties and challenges of targeting treatment especially for patients with WAD II, which is the most prevalent group of WAD severity [95]. Taking our findings into account, the current grading system likely oversimplifies a heterogenous group of patients which may require distinct treatment approaches.

## Clinical implications

This review suggests that not all patients may fit the traditionally defined categories of WAD I–IV [71]. As we identified dysfunction in both the large and small nerve fibres, a comprehensive clinical neurological examination extending beyond the traditional light touch, muscle strength and reflex testing and including small fibre tests (e.g., thermal thresholds) is critical for these patients. Small fibre pathology has been shown to precede findings of inherent large fibre pathology in patients with focal nerve injury [86; 87], but this remains to be shown for patients with WAD. Furthermore, we may have to consider the sensitivity of the traditional neurological examination in detecting sensory loss. Our findings suggest that quantitative sensory testing methods demonstrate dysfunction in patients who are classified as having no neurological deficit upon routine clinical neurological examination (WAD II). It remains to be explored whether more sensitive detection of sensory changes impacts the prediction of patient outcomes or choice of intervention. Importantly, sensory changes in patients with WAD must be interpreted in the context of a careful clinical examination, taking other mechanisms such as nociplastic changes into account.

An incomplete clinical assessment may also create dissonance between subjective reports of neuropathic symptoms that lack corresponding objective findings. Qualitative reports of patient challenges highlight difficulties with feeling understood or properly treated, which contribute to prolonged distress and trauma [81]. Similarly, some patients reported their WAD symptoms did not match the management strategies suggested by their healthcare provider [82]. Including a detailed evaluation may improve personal patient challenges and may also help direct more targeted management strategies.

Importantly, the management of neuropathic pain differs from nociceptive pain [27]. Current treatment guidelines for WAD II do not include management strategies for nerve-related pathology or neuropathic pain [1; 31]. Our findings suggest that this may need to be considered for a subset of patients. There are currently several efforts underway to examine the benefit of targeted neuropathic treatments for patients with WAD [39; 70] and results from preliminary studies may be promising [70]. Such studies are required to determine whether interventions targeting neuropathic pain and nerve pathology may be beneficial in a subset of patients.

## Limitations

The primary limitations of this study are the overall risk of bias and some data heterogeneity. Many studies had a risk of bias, which was often due to small sample sizes and comparability of selected outcome groups. High data heterogeneity was seen in some meta-analyses, particularly regarding nerve mechanosensitivity. It is also important to consider potential publication bias. Negative findings for nerve pathology and neuropathic pain might be less likely to be reported. Lastly, limitations in generalisability involve the inclusion of only English language articles, single author screening for initial abstract eligibility, and that some meta-analyses included studies from only one research group.

## Conclusions

Our data suggest that nerve pathology and signs of neuropathic pain are present in a subset of patients after whiplash injury. Importantly, this included patients categorised as WAD grade II, who are traditionally classified by the lack of neurological signs. Therefore, including detailed clinical assessments and clinical screening for neuropathic pain and nerve pathology is recommended for patients with WAD. Future research including large prospective cohorts is needed to identify underlying mechanisms of nerve pathology and neuropathic pain and to evaluate whether targeting treatments at neuropathic pain and nerve pathology improves clinical outcomes of this specific subgroup of patients with whiplash injuries.

## Supplementary Material

Supplemental tables

## Figures and Tables

**Table 1:** Study characteristics and outcome measures

Author & Date	Study Participants	WAD Grade (QTF)	Study Measures	Outcomes: mean (SD)	
**Sterling (2009)^106^**	WAD n=85	I-III	S-LANSS (0-24)	n=12 patients scoring ≥12	
**Smith (2013)^90^**	WAD n=90, controls n=30	II	S-LANSS (0-24)	WAD (R; n=58): 11 (IQR 8-17), (NR; n=32): 13 (IQR 8-16)	
			ULNT (degrees of elbow flexion)*	WAD (R): 29.33 (16.30), NR: 34 (14.81)	Controls: 4 (6.67)
			PPT (kPa)*	WAD (R): 236 (78.15), WAD (NR): 229.34 (85.92)	Controls: 375.17 (134.38)
**Karlsborg (1997)^58^**	WAD n=34	II-IV	Neurological Examination	n=5 patients (positive findings of nerve pathology)	
**Henrikson (2013)^53^**	WAD n=20, controls n=10	II-III	Thermal detection (Thermotest)	No group mean values provided. n=6 with reduce temperature sensitivity; n=5 with increased temperature sensitivity (facial skin)	
**Chuang (2002)^20^**	WAD n=85	NA	Electrodiagnostic testing	n=7 patients (positive findings of nerve pathology)	
**Smith (2014)^92^**	WAD n=53, controls n=30	II	ULNT (degrees of elbow flexion)*	WAD: 28.67 (15.56)	Controls: 4 (6.67)
			PPT (kPa)*	WAD: 185.33 (63.70)	Controls: 365.33 (98.27)
**Sterling (2004)^104^**	WAD n=80, controls n=20	II-III	ULNT (degrees of elbow flexion)	mild: 26.7 (17.7), moderate: 31.3 (14.9), severe: 36.5 (11.8)	Controls: 21.4 (10.8)
**Serrano-Munoz (2019)^89^**	WAD n=20, control n=15	I-III	DN4 (0-10)	No Pain (n=5): median 3 (IQR 1.5), Pain (n=15): median 4 (IQR 3) out of 10.	
			NPSI (0-10)	No Pain (n=5): median 0 (IQR 2), Pain (n=15): median 3 (IQR 6) out of 10.	
**Sterling (2010)^96^**	WAD n=62, controls n=22	II-III	PPT (kPa)	(R) 197.6 (71) Mild: 220 (77) Moderate/severe: 140.3 (77) (measures from 3 weeks)	Controls: 235 (70) (time 1)
**Bowles (2004)^11^**	WAD n=25	NA	Electrodiagnostic testing	N=25 patients (positive findings of nerve pathology)	
**Greening (2018)^44^**	WAD n=9, controls n=13	II	S-LANSS (0-24)	Mean: 12.7 (7.5) n=4 scores ≥12	
			ULNT	Symptomatic side: median n=9, ulnar n=6; Less symptomatic side: median n=4, ulnar n=1 (symptom reproduction)	
			Neurologic examination	n=4 (44.4%) reduced cutaneous sensation in median nerve	
			Magnetic resonance imaging	Greater T2 signal intensity (brachial plexus, median nerve -wrist): WAD mean= 0.52 ± 0.13 and 2.09 ± 0.33, respectively) compared to the control group (mean= 0.45 ± 0.07 and 1.38 ± 0.31, respectively; p<.05).	
			Nerve palpation	symptomatic side: brachial plexus n=8, median nerve n=5, cubital tunnel n=7, Guyon’s canal n=6 less symptomatic side: brachial plexus n=4, median nerve n=1, cubital tunnel n= 3, Guyon’s canal n=2 (+ for local or referred pain &/or paresthesia)	
**Hashish (2017)^50^**	WAD n=903	NA	Electrodiagnostic testing	cervical radiculitis: n= 315; lumbar radiculitis n= 216	
**Chien (2009)^17^**	WAD n=31, controls n=31	II	ULNT (degrees of elbow flexion)	WAD: 22.3 (27.4)	Controls: 11.0 (5.9)
			PPT (kPa)	WAD: 212.67 (99.17)	Controls: 00.97 (61.26)
			Thermal detection thresholds (°C)	WAD heat index finger: 34.91 (2.29), little finger: 34.43 (2.2), Cold index finger: 28.99 (1.55), little finger: 28.62(2.05)	Controls heat index finger: 32.35 (1.43), little finger: 32.32 (1.12), Cold index: 29.58 (.85), little finger: 29.56 (.82)
			Vibration detection thresholds (μm)	WAD dorsal 5th: 0.48 (0.4), dorsal 2nd: 0.4 (0.27), palmar 2nd: 0.46 (0.31), palmar 1st: 0.79 (0.62)	Controls dorsal 5th: 0.29 (0.12), dorsal 2nd: 0.26 (0.09), palmar 2nd: 0.28 (0.16), palmar 1st: 0.41 (0.25)
			Current detection threshold 2,000 Hz (mA)	WAD elbow: 106.9 (26.64), index finger: 254.44 (55.84), little finger: 193.53 (40.96), tibialis anterior: 186.92 (78.15)	Controls elbow: 88.82 (22.33), index finger: 180 (45.08), little finger: 145.46 (31.88), tibialis anterior: 151.52 (56.24)
			Current detection threshold 250 Hz (mA)	WAD elbow: 41.84 (34.1), index finger: 84.79 (32.23), little finger: 83.65 (40.31), tibialis anterior: 37.26 (14.64)	Controls elbow: 32.61 (8.68), index finger: 62.16 (25.88), little finger: 60.5 (21.89), tibialis anterior: 41.94 (14.45)
			Current detection threshld 5 Hz (mA)	WAD elbow: 22 (9.15), index finger: 46.35 (20.49), little finger: 42.53 (25.79), tibialis anterior: 27.89 (17.38)	Controls elbow: 22.16 (10.15), index finger: 35.23 (16.36), little finger: 34.84 (14.02), tibialis anterior: 23.11 (10.03)
**Chien (2008b)^15^**	WAD n=52, controls n=31	NA	ULNT (degrees of elbow flexion)	WAD high risk: 51.65 (21.15), low risk: 29.72 (21.83)	Controls: 11.62 (5.96)
			PPT (kPa)	WAD high risk: 173.21 (68.45), low risk: 246.66 (91.57)	Controls: 300.97 (61.26)
			Thermal detection thresholds (°C)	WAD index finger heat: low risk 32.65 (1.42), High risk 32.78 (1.98) Little finger heat: low risk 33.20 (1.94), high risk 33.14 (2.10) Index finger cold: low risk 28.93 (0.75), high risk 28.73 (0.84), little finger cold: low risk 28.68 (0.90), high risk 28.63 (0.93)	Controls index finger heat: 32.35 (1.43), little finger heat: 32.32 (1.12) Index finger cold: 29.32 (0.52), little finger cold: 29.29 (0.50)
			Vibration detection thresholds (μm)	WAD dorsal 5th: low risk 0.41 (0.24), high risk 0.56 (0.57). Palmar 2nd: low risk 0.38 (0.23), high risk 0.56 (0.69). Palmar 1st: low risk 0.51 (0.33), high risk 0.64 (0.54)	Controls dorsal 5th: 0.29 (0.12), Palmar 2nd: 0.28 (0.16), Palmar 1st: 0.41 (0.25)
			Current detection thresholds 250 Hz (mA)*	WAD elbow: low risk 40.96 (10.19), high risk 50.44 (29.62) Index finger: low risk 101.16 (26.84), high risk 124.88 (50.59) Little finger: low risk 99.14 (22.59), high risk 125.09 (82.12), Tibia: low risk 80.23 (24.62), high risk 93.68 (60.96)	Controls elbow: 32.61 (8.68), index finger: 62.16 (25.88), little finger: 58.82 (22.40), tibia: 41.94 (14.45)
**Vaegter (2018)^119^**	WAD n=108	NA	Warm detection thresholds (°C)	WAD PTSD group = 34.0 (1.3) WAD Non-PTSD group = 34.7 (1.1)	
**Greening (2005)^45^**	WAD n=9, controls n=8	NA	ULNT (degrees of elbow flexion)	WAD: n=9 (positive for symptom reproduction)	Controls: n=0 (positive for symptom reproduction)
			Nerve palpation	WAD carpal tunnel: n=6, proximal carpal tunnel n=5, brachial plex n=7 (positive for symptom reproduction)	Controls: n=0 (positive for symptom reproduction)
			Ultrasound	WAD: significantly reduced longitudinal (mean=0.38 (0.08) mm, (95% CI=0.20–0.56 mm)) and transverse nerve movement (2.57 (0.80) mm, (95% CI=0.61–4.54 mm)) on the symptomatic side compared to the control group.	
**Pedler (2013)^75^**	WAD n=64, controls n=24	I-II	PPT (kPa)	No group mean values provided. Reported significant difference between left and right sides for median nerve PPT (p<0.01)	
**Radanov (1995)^80^**	WAD n=117	I-III	Neurological examination	n=17 (tests positive findings of nerve pathology)	
**Alpar (2002)^2^**	WAD n=38, controls n=30	NA	Neurological examination	n=38 with hypoaesthesia to light touch and pin prick (median nerve distribution)	
			Nerve palpation	n=36 (positive symptom reproduction)	
			Electrodiagnostic testing	n=11 patients had abnormal EMG and NCV results	
**Pettersson (1994)^76^**	WAD n=39	NA	Neurological examination	Trigeminal nerve hypoaesthesia n=9 Reduced myotomal strength n=4 UE hypoaesthesia light touch n=15 UE hyporeflexia n=6 At least one abnormal finding n=19	
**Midha (1997)^67^**	WAD n=16	NA	Electrodiagnostic testing	n=16 positive studies for nerve pathology	
**Miranda (2016)^68^**	WAD n=20	NA	Electrodiagnostic testing	n=20 positive studies for nerve pathology	
**Jonsson (1994)^55^**	WAD n=24	NA	Neurological examination	n=19 patients (positive neurologic findings)	
**Braddom (2009)^12^**	WAD n=1,334	NA	Electrodiagnostic testing	n=1,248 positive for nerve pathology	
**Kaiser (2014)^56^**	WAD n=12	NA	Electrodiagnostic testing	n=12 positive for nerve pathology	
**Coert (1994)^22^**	WAD n=157	NA	Electrodiagnostic testing	n=157 total positive for nerve pathology carpal tunnel syndrome=68, cubital tunnel syndrome = 64, radial sensory nerve = 25	
**Sterling (2006b)^101^**	WAD n=65	II-III	PPT (kPa)	Median nerve (recovered, mild, moderate/severe; >1 month): 197.6 (70.6), 231.8 (65.1), 210.5 (74.7) 6 months: 244 (64.6); 140.9 (50.5), 169.9 (54.7)	
			Sympathetic vasoconstrictor reflex	QI (recovered, mild, moderate/severe; >1 month): 58.4 (17.2), 55.7 (16.9); 52.19 (16.9) 6 months: 56.1 (15); 69.68 (18.2), 69.44 (17) SRF (recovered, mild, moderate/severe; >1 month): 0.75 (0.17), 0.75 (0.2); 0.76 (0.18) 6 months: 0.76 (0.17); 0.61 (0.15), 0.63 (0.14)	
**Sterling (2005)^104^**	WAD n=76	II-III	PPT (kPa)	Recovered, mild, moderate/severe, (>1 month): 197.6 (70.6), 210.5 (74.7), 140.9 (50.5) Recovered, mild, mod/severe, (6 months): 231.8 (65.1); 244 (64.6); 169.9 (54.7)	
			Sympathetic vasoconstrictor reflex	QI (recovered, mild, moderate/severe; >1 month): 58.4 (17.2), 52.19 (16.9), 69.68 (18.2) 6 months: 55.7 (16.9); 56.1 (15); 69.44 (17) SFR (recovered, mild, moderate/severe; >1 month): 0.75 (0.17), 0.76 (0.18), 0.61 (0.15) 6 months: 0.75 (0.2); 0.76 (0.17); 0.63 (0.14)	
**Sturzenegger (1994)^109^**	WAD n=137	I-III	Neurological examination	N=17 patients (positive findings of nerve pathology)	
**Goudman (2020)^43^**	WAD n=21, controls n=18	I-III	Laser evoked potential	WAD hand (amplitudes,μV): n1: -4.67 (2.81); N2: -2.54 (1.70), P2: 4.27 (3.11), N2P2: 6.81 (4.32) Latency (msec): N1: 224 (53), N2 (225 (50), P2: 388 (70)	Controls hand (amplitudes,μV): N1 -4.47 (2.37), N2: -3.41 (3.25), P2: 5.56 (2.83), N2P2: 8.97 (5.29) Latency (msec): N1: 252 (56), N2: 229 (54), P2: 374 (59)
**Sterner (2001)^108^**	WAD n=43	NA	Thermal detection threshold	n=14 patients with abnormal results (trigeminal nerve)	
			Vibration detection threshold	n=11 patients with abnormal results (trigeminal nerve)	
**Radanov (1994)^79^**	WAD n=117	NA	Neurological examination	N=17 patients (positive findings of nerve pathology)	
**Sterling (2002)^107^**	WAD n=156, controls n=95	II-III	ULNT (degrees of elbow flexion)*	WAD: 26.21 (11.73)	Controls: 12.92 (14.78)
			Neurological examination	n=23 patients (positive findings of nerve pathology)	
**Bekelis (2014)^7^**	WAD n=384,539	NA	ICD-9 codes	n=3,086 patients (peripheral nerve injury)	
**Lo (2007)^62^**	WAD n=20	I-III	Neurological examination	n=10 patients (positive findings of nerve pathology)	
			Electrodiagnostic testing	n=2 patients (positive findings of nerve pathology)	
			Cutaneous silent period	n=18 patients with abnormal findings of at least one recording (measured at hand and foot).	
**Sterling (2003)^102^**	WAD n=76, controls n=20	II-III	ULNT (degrees of elbow flexion)*	WAD: 26.21 (11.73)	Controls: 12.92 (14.78)
			Sympathetic vasoconstrictor reflex*	WAD recovered QI: 54 (149.98), SRF: 0.79 (1.48) mild QI: 53.1 (147.37), SRF: 0.79 (1.57), mod/severe QI: 64.8 (158.70), SRF: 0.69 (1.31)	Controls QI: 52.3 (82.25) SRF: 0.71 (0.80)
**Chien (2010)^18^**	WAD n=50, controls n=31	II	Thermal detection threshold (°C)*	WAD heat detection index finger: 34.93 (4.81), little finger: 34.70 (4.94) Cold detection index finger: 28.24 (1.20), little finger: 27.80 (1.98)	Controls heat detection index finger: 32.32 (3.29) little finger: 32.32 (3.73) Cold detection index finger: 29.30 (3.23), little finger: 29.28 (3.28)
			Vibration detection threshold (μm)*	WAD palmar 1st 0.83 (0.92), palmar 2nd: 0.54 (0.57), dorsal 5th: 0.51 (0.57)	Controls palmar 1st: 0.41 (0.45), palmar 2nd: 0.28 (0.33), dorsal 5th: 0.42 (0.45)
			Current detection threshold 250 Hz (mA)*	WAD elbow: 46.93 (87.60), index finger: 94.27 (170.46), little finger: 87.06 (159.08), tibialis anterior: 44.30 (79.04)	Controls elbow: 32.48 (29.97), Index finger: 32.38 (34.53), little finger: 58.88 (59.26), tibialis anterior: 41.84 (38.32)
			PPT (kPa)	WAD: 187.9 (87.9)	Controls: 301.0 (45.0)
**Farrell (2020)^32^**	WAD n=24, controls n=24	II	S-LANSS (0-24)	7.5 (6.5)	
			NPSI (0-100)	26.1 (18.3)	
			Neurological examination	N=0 patients (positive for nerve pathology)	
			Thermal detection threshold (°C)	WAD cold index finger: 30.17 (1.16), warm detection index finger: 35.02 (1.55)	Controls cold index finger: 30.75 (0.36), warm index finger: 33.85 (0.47)
			Vibration detection threshold (disappearance)	WAD index: 7.88 (0.27)	Controls index: = 7.96 (0.16)
			Mechanical pain threshold (mN)	WAD index: 205.42 (142.47)	Controls index: 161.68 (96.41)
			Mechanical detection threshold (mN)	WAD index: 1.06 (0.82)	Controls index: 0.48 (0.18)
			Intraepidermal nerve fibre density (fibres/mm)	WAD index finger (median (IQR)): 4.5 (4.9) WAD ankle: 7.3 (3.7)	Controls index (median (IQR)): 7.3 (3.9) Ankle: 9.3 (3.8)
			Dermal innervation	WAD index finger (median (IQR)): 3.7 (2.8) bundles/mm^2^ Meissner corpuscles density: (median (IQR)): 0.41 (0.51) corpuscles/mm	Controls index finger (median (IQR)): 4.9 (2.1) bundles/mm^2^ Meissner corpuscles density: (median (IQR)): 0.61 (0.52) corpuscles/mm
**Squires (1996)^92^**	WAD n=37	NA	Neurological examination	n=4 patients (positive for nerve pathology)	
**Chuang (1998)^19^**	WAD n=14	NA	Electrodiagnostic testing	n=14 patients (positive for nerve pathology)	
**Sturzenegger (1995)^110^**	WAD n=117	NA	Neurological examination	n=17 patients (positive for nerve pathology)	
**Saadat (2011)^83^**	WAD n=78	NA	ICD-9 codes	n=78 patients (positive peripheral nerve injury)	
**Moog (2002)^69^**	WAD n=43, controls n=43	I-II	Sensation detection	n=0 patients with inability to detect light touch, punctate pressure, warm and cold detection	
			Vibration detection	No mean values provided. All participants reported detection within 10-15% of available frequency.	
**Sterling (2006a)^105^**	WAD n=76	I-III	PPT (kPa)*	Median nerve (mean/SEM): <1 month, PTSR: 155.12 (80.82), resPTSR: 187.55 (105.77), nonPTSR: 201.72 (76.25) 6 months, PTSR: 166.53 (82.12), resPTSR: 242.78 (76.25), nonPTSR: 230.56 (74.79)	
			Sympathetic vasoconstrictor reflex*	QI (<1 month), PTSR: 70.78 (19.62), resPTSR: 59.75 (20.1), nonPTSR: 55.42 (19.61) QI (6 months), PTSR: 70.66 (19.14), resPTSR: 57.48 (19.86), nonPTSR: 57.48 (19.86) SFR (<1 month), PTSR: 0.56 (.20), resPTSR: 0.70 (.20), nonPTSR: 0.75 (0.20) SFR (6 months), PTSR: 0.60 (0.20), resPTSR: 0.74 (0.20), nonPTSR: 0.74 (0.20)	
**Wallin (2012)^121^**	WAD n=28,	II-III	Thermal detection threshold (°C)*	WAD cold: thenar 29.8 (1.3), trapezius=29.8 (2.3), tibialis anterior= 28.2 (3.6) Warm thenar: 34.1 (2.0), trapezius: 35.9 (2.5), tibialis anterior: 37.8 (4.7)	Controls cold: thenar: 30.7 (0.4), trapezius=30.9 (1.4), tibialis anterior=29.1 (1.4) Warm thenar: 33.5 (0.4), trapezius: 35.1 (1.4), tibialis anterior: 37.2 (2.7)
**Raak (2006)^78^**	WAD n=17, controls n=18	NA	Thermal detection threshold (°C)	WAD thenar warm: 35.03 (2.67), cold 29.42 (2.12). trapezius warm 37.94 (4.39), cold 28.75 (4.94)	Controls thenar warm: 33.71 (0.57), cold: 29.88 (1.26). trapezius warm: 35.80 (3.13), cold: 30.28 (1.17)
**Mailis (1995)^65^**	WAD n=32	NA	Nerve palpation	N=32 patients (positive for symptom reproduction upon pressure)	
**Kaiser (2012)^57^**	WAD n=75	NA	Electrodiagnostic testing	n=75 studies (positive for nerve injury)	
**Chien (2008a)^16^**	WAD n=50, controls n=31	II	ULNT (degrees of elbow flexion)	WAD: 21.3 (25.5)	Controls: 11.0 (5.21)
			Thermal detection threshold (°C)*	WAD (mean, 95% CI), heat, index finger: 34.91 (34.05, 35.63), little finger: 34.71 (33.78, 35.63) Cold index finger: 28.27 (27.32, 29.22), little finger: 27.82 (26.75, 28.90)	Controls (mean, 95% CI), heat, index finger: 32.35 (31.83, 32.88), Little finger: 32.32 (31.91, 32.73) Cold index finger: 29.32 (29.13, 29.51), little finger: 29.29 (29.10, 29.47)
			Current detection threshold 250 Hz (mA)*	WAD (mean, 95% CI) elbow: 47.13 (36.24, 58.02). index finger: 94.15 (80.78, 107.52) little finger: 86.81 (74.98, 98.64) tibialis anterior: 44.43 (37.03, 51.83)	Controls (mean, 95% CI) elbow: 32.61 (29.43, 35.80), index finger: 62.16 (52.67, 71.65), little finger: 58.82 (50.61, 67.04), tibialis anterior: 41.94 (36.64, 47.24)
			Vibration detection threshold (μm)’	WAD (mean, 95% CI): palmar 1st: 0.83 (0.64, 1.02), palmar 2nd: 0.54 (0.38, 0.65), dorsal 5th: 0.51 (0.36, 0.65)	Controls (mean, 95% CI): palmar 1st: 0.41 (0.32, 0.50),palmar 2nd: 0.28 (.022, 0.34), dorsal 5th 0.29 (0.43, 0.71)
			PPT (kPa)*	WAD (mean, 95% CI): 196.00 (171.35, 220.66)	Controls (mean, 95% CI): 300.97 (278.5, 323.44)
**Maimaris (1988)^66^**	WAD n=102	NA	Neurological examination	n=18 patients (positive for nerve pathology)	
**Ovadia (2002)^73^**	WAD n=866	NA	Neurological examination	n=20 patients (positive for nerve pathology)	
			Electrodiagnostic testing	n=127 studies with abnormal findings (EMG)	
**Steinberg (2005)^94^**	WAD n=330	I-II	Electrodiagnostic testing	n=104 studies with abnormal findings (EMG)	
**Terzis (2009)^115^**	WAD n=25	NA	Electrodiagnostic testing	n=25 studies positive test for nerve pathology	
**Scott (2005)^88^**	WAD n=29	II	PPT (kPa)*	WAD median: 162.68 (243.90) ulnar: 281.55 (263.94) radial: 191.04 (243.90)	Controls median: 274.55 (255.47) ulnar: 373.22 (285.19) radial: 296.80 (232.17)

* mean/sd estimated from graph or transformed from alternatively reported summary statistic. **Abbreviations:** (NR): non-recovered; (R): recovered; DN4: Douleur Neuropathique 4; EMG: electromyography; ICD: International Classification of Diseases; IQR: interquartile range; NCV: nerve conduction velocity; nonPTSR: non-posttraumatic stress reaction; NPSI: Neuropathic Pain Symptom Inventory; PPT: pressure pain threshold; PTSD: Posttraumatic Stress Disorder; PTSR: Posttraumatic stress reaction; QI: quotient interval; QTF: Quebec Task Force; NA: not available; resPTSR: resolved posttraumatic stress reaction; S-LANSS: Self-complete Leeds Assessment of Neuropathic Symptoms and Signs; SEM: standard error of the mean; SRF: sympathetic reflex; UE: upper extremity; ULNT: upper limb neurodynamic test: WAD: whiplash associated disorders.

**Table 2:** Certainty of neuropathic pain at study level according to the IASP Neuropathic Pain Grading System.

	Possible	Probable	Definite	Outcome
Article	History neurologic lesion & neuroanatomically plausible	Sensory signs	Diagnostic tests	
**Sterling 2009**	Patients after whiplash injury reporting neck pain, S-LANSS (34% positive)	NA	NA	Possible
**Smith 2013**	Patients after whiplash injury reporting neck pain, S-LANSS (36% positive)	NA	NA	Possible
**Karlsborg 1997**	Patients after whiplash injury reporting neck pain	n=5/34 patients with upper extremity sensory loss (light touch)	NA	Probable
**Henrikson 2013**	Patients after whiplash injury reporting neck pain	n=5/20 reduced temperature sensitivity	NA	Probable
**Chuang 2002**	Included patients after whiplash injury measuring the brachial plexus	NA	n=7/85 positive NCV and EMG findings of nerve pathology	Nerve pathology
**Smith 2014**	Patients after whiplash injury reporting neck pain, 21% reporting upper extremity symptoms	NA	NA	Possible
**Sterling 2004**	Patients after whiplash injuring reporting neck pain	NA	NA	Possible
**Serrano-Munoz 2019**	Pain after whiplash injury, DN4 (n=15/20 indicating neuropathic pain), and NPSI questionnaires (median score pain group: 3/10)	NA	NA	Possible
**Sterling 2010**	Patients after whiplash injuring reporting neck pain, 48% reporting upper limb symptoms	NA	NA	Possible
**Bowles 2004**	Included patients after whiplash injury measuring the brachial plexus	NA	n=25/25 patients with positive EMG findings of nerve pathology	Nerve pathology
**Greening 2018**	Patients after whiplash injury reporting painful symptoms in upper limb, S-LANSS questionnaire (n=4/9 patients indicating neuropathic pain)	n=4/9 (44.4%) reduced cutaneous sensation in median nerve	MRI: increased T2 signal intensity brachial plexus and median nerve at wrist	Definite
**Hashish 2017**	Patient after whiplash injury referred to pain clinic for cervical and lumbar nerve assessment	NA	Positive EMG testing: cervical radiculitis: n= 315/903; lumbar radiculitis n= 216/903	Nerve pathology
**Chien 2009**	Patients after whiplash injury reporting neck pain, 45% reporting arm pain	Abnormal upper extremity thermal, vibration, and current detection thresholds	NA	Probable
**Chien 2008b**	Patients after whiplash injury reporting neck pain	Abnormal upper extremity thermal, vibration, and current detection thresholds	NA	Probable
**Vaegter 2018**	Patients after whiplash injury reporting spinal pain	Abnormal upper extremity warm detection thresholds	NA	Probable
**Greening 2005**	Patients after whiplash injury reporting neck and arm pain	NA	NA	Possible
**Pedler 2013**	Patients after whiplash injury reporting pain	NA	NA	Possible
**Radanov 1995**	Patients with pain after whiplash injury, 49% reporting shoulder pain, 92% reporting neck pain, 15% reporting dermatomal paraesthesia, tingling	n=17/117 patients neurologic deficit (sensory loss, reflex loss, paresis)	NA	Probable
**Alpar 2002**	Patients after whiplash injury reporting neck and shoulder pain	n=38/38 with hypoaesthesia to light touch and pin prick (median nerve distribution)	n=11/38 patients had abnormal EMG and NCV results	Definite
**Pettersson 1994**	Patients after whiplash injury reporting neck pain, 69% shoulder pain	Trigeminal nerve hypoaesthesia n=9/39 Reduced myotomal strength n=4/39 UE hypoaesthesia light touch n=15/39 UE hyporeflexia n=6/39	NA	Probable
**Midha 1997**	Included patients after whiplash injury measuring the brachial plexus	NA	n= 16/16 abnormal EMG or NCV results	Nerve pathology
**Miranda 2016**	Included patients after whiplash injury	NA	n=20/20 abnormal EMG and NCV studies for nerve pathology	Nerve pathology
**Jonsson 1994**	Patients after whiplash injury reporting neck pain, 79% radiating arm pain	n=19/24 patients with decreased strength, sensation, or reflexes	NA	Probable
**Braddom 2009**	Patients after whiplash injury referred to pain clinic for cervical and lumbar nerve assessment	NA	n=1,248/1,334 abnormal EMG studies for nerve pathology	Nerve pathology
**Kaiser 2014**	Included patients after whiplash injury measuring the brachial plexus	NA	n=12/12 abnormal EMG and NCV studies for nerve pathology	Nerve pathology
**Coert 1994**	Included patients after whiplash injury measuring the median, radial, or ulnar nerves	NA	n=157/157 abnormal EMG or NCV for nerve pathology	Nerve pathology
**Sterling 2006b**	Patients after whiplash injury reporting neck pain, 20% reported shoulder pain	NA	NA	Possible
**Sterling 2005**	Patients after whiplash injury reporting neck pain, 30% reporting shoulder pain	NA	NA	Possible
**Sturzenegger 1994**	Patients after whiplash injury reporting pain, 35% reported neurologic symptoms, 49% reported shoulder pain	N=17/137 patients had neurologic deficit (sensory loss, reflex loss, or paresis with radicular distribution)	NA	Probable
**Goudman 2020**	Patients after whiplash injury reporting pain	NA	No significant differences in laser evoked potentials	Possible
**Sterner 2001**	Patients after whiplash injury reporting pain, 47% report radiating pain in arms and hands, 59% report paraesthesia in arms and hands	n=14/43 patients abnormal thermal detection and n=11/43 patients abnormal vibration detection (trigeminal nerve)	NA	Probable
**Radanov 1994**	Patients after whiplash injury reporting neck pain, 49% reported shoulder pain	N=17/117 patients neurologic deficit in radicular pattern (weakness, hyporeflexia, or hypoaesthesia)	NA	Probable
**Sterling 2002**	Patients after whiplash injury reporting pain	n=23/156 patients (weakness, hyporeflexia, or hypoaesthesia)	NA	Probable
**Bekelis 2014**	Patients after whiplash injury, n=3,086 patients ICD-9 codes for peripheral nerve injury	NA	NA	Possible
**Lo 2007**	Patients after whiplash injury reporting neck pain	n=10/20 patients (weakness, hyporeflexia, or hypoaesthesia)	n=2/20 with abnormal EMG testing, n=18 with at least one abnormal recording of cutaneous silent periods	Definite
**Sterling 2003**	Patients after whiplash injury reporting neck pain	NA	NA	Possible
**Chien 2010**	Patients after whiplash injury reporting neck pain, 45% reported radiating arm pain	Reduced thermal, vibration, and current detection thresholds	NA	Probable
**Farrell 2020**	Patients after whiplash injury reporting neck pain, 46% shoulder or arm pain, 17% forearm or hand pain, S-LANSS and NPSI questionnaires	N=0/24 patients with abnormal strength, reflexes, and light touch sensation. Findings of reduced thermal and mechanical pain thresholds	Reduced dermal and intraepidermal nerve fibre density (skin biopsy)	Definite
**Squires 1996**	Patients after whiplash injury reporting pain, 45% report paraesthesia	n=4/37 patients (weakness, hyporeflexia, or hypoaesthesia)	NA	Probable
**Chuang 1998**	Included patients after whiplash injury measuring the brachial plexus	NA	n=14/14 abnormal EMG and NCV studies for nerve pathology	Nerve pathology
**Sturzenegger 1995**	Patients after whiplash injury reporting neck pain	n=17/117 patients (weakness, hyporeflexia, or hypoaesthesia)	NA	Probable
**Saadat 2011**	Included patients after whiplash injury, n=78 patients positive for peripheral nerve injury using ICD-9 codes	NA	NA	Possible
**Moog 2002**	Patients after whiplash injury reporting pain	n=0/43 patients with inability to detect light touch, punctate pressure, warm and cold detection	NA	Possible
**Sterling 2006a**	Patients after whiplash injury reporting neck pain	NA	NA	Possible
**Wallin 2012**	Patients after whiplash injury reporting neck and shoulder pain	Reduced thermal detection thresholds	NA	Probable
**Raak 2006**	Patients after whiplash injury reporting pain	Reduced thermal detection thresholds	NA	Probable
**Mailis 1995**	Patients after whiplash injury reporting pain, 84% reported paraesthesia	NA	NA	Possible
**Kaiser 2012**	Included patients after whiplash injury measuring the brachial plexus	NA	n=75/75 abnormal EMG or NCV studies for nerve injury)	Nerve pathology
**Chien 2008a**	Patients after whiplash injury reporting neck pain, 45% reported radiating arm pain	Reduced thermal, vibration, and current detection thresholds	NA	Probable
**Maimaris 1988**	Patients after whiplash injury reporting pain, 46% reported shoulder pain	n=18/102 patients (weakness, hyporeflexia, or hypoaesthesia)	NA	Probable
**Ovadia 2002**	Patients after whiplash injury reporting pain, 25% reporting shoulder pain, 36% reporting upper limb pain	n=20/866 patients (weakness, hyporeflexia, or hypoaesthesia)	n=127/866 with abnormal EMG findings	Definite
**Steinberg 2005**	Patients after whiplash injury reporting pain, 7% reported radiating shoulder pain	NA	n=104/330 with abnormal EMG findings	Nerve pathology
**Terzis 2009**	Included patients with pain after whiplash injury measuring the brachial plexus	NA	n=25/25 abnormal NCV and EMG findings for nerve pathology	Nerve pathology
**Scott 2005**	Patients after whiplash injury reporting pain	NA	NA	Possible

**Abbreviations:** Douleur Neuropathique 4 (DN4), Electromyography (EMG), Not available (NA), Nerve conduction velocity (NCV), Neuropathic Pain Symptom Inventory (NPSI), Self-complete Leeds Assessment of Neuropathic Symptoms and Signs (S-LANSS). *Nerve pathology indicates studies that reported outcomes of diagnostic tests confirming a lesion of the somatosensory nervous system (definite neuropathic pain) but did not report sensory signs (probable neuropathic pain)*.
