# Supplementary material for: Nerve pathology and neuropathic pain after whiplash injury: a systematic review and meta-analysis
Source: Pain. Author manuscript; Available in PMC 2022 Jul 1. (PMC7612893; doi:10.1097/j.pain.0000000000002509)
Supplement: Supplemental tables [file EMS137227-supplement-Supplemental_tables.pdf]

Supplemental Table S1. Example search strategies for all included search engines.

Pubmed

S1	(MH “Whiplash”) OR (MH “neck injuries)
S2	(MH Cohort studies) OR (MH Case-control studies) OR (MH observational study) OR (MH Cross-sectional studies)
S3	S1 AND S2
S4	(MH peripheral nervous system disease) OR (MH somatosensory disorder) OR (MH neuropathic pain) OR (MH nerve disorder)
S5	(MH magnetic resonance imaging) OR (MH diffusion tensor imaging) OR (MH ultrasonography)
S6	(‘painDETECT’ OR ‘Douleur Neuropathique en 4 question’ OR ‘DN4’ OR ‘S-LANSS’ OR ‘Neuropathic Pain Symptom Inventory’ OR ‘Neuropathic Pain Questionnaire’ OR ‘ID Pain’ OR ‘Neuropathic Pain Scale’)
S7	(MH quantitative sensory testing) OR (neurological examination) OR (MH muscle strength) OR (MH electrodiagnosis) OR (MH neural conduction) OR (‘dermatome OR ‘myotome’) OR (sympathetic reflexes) OR (small fibre neuropathy OR small fiber neuropathy OR small fibre sensory neuropathy OR small fiber sensory neuropathy OR small fibre pathology OR small fiber pathology) OR (intraneural change)
S8	(MH prevalence) OR (MH incidence) OR (MH epidemiology)
S9	S3 AND S4
S10	S3 and S5
S11	S3 AND S6
S12	S3 AND S7
S13	S3 AND S4 AND S8

Embase and MEDLINE

1	'whiplash'/exp OR 'traffic accident'/exp OR 'whiplash associated disorder'/exp
2	'cohort study'/exp OR 'case control study’/exp OR 'observational study'/exp OR 'cross-sectional study'/exp
3	1 and 2
4	'peripheral nervous system disease' OR 'somatosensory disorder'/exp OR 'neuropathic pain'/exp OR 'nerve disorder' OR 'neuralgia'/exp OR 'peripheral nerve injury'/exp OR 'cervical plexus'/exp OR 'spinal nerve roots'/exp
5	‘neurological exam’/exp OR ‘muscle strength’/exp OR 'electrodiagnosis’/exp OR ‘neural conduction’ OR ‘dermatome’ OR ‘myotome’ OR ‘sympathetic reflex’ OR 'small fibre neuropathy’ OR ‘small fiber neuropathy’ OR ‘small fibre sensory neuropathy’ OR ‘small fiber sensory neuropathy’ OR 'small fibre pathology’ OR ‘small fiber pathology’ OR ‘intra neural change’ OR 'quantitative sensory testing’ OR ‘muscle weakness’ OR ‘sensory testing’
6	'douleur neuropathique 4 questions' OR 'leeds assessment of neuropathic symptoms and signs' OR 'paindetect' OR 'dn4' OR 'lanss' OR 's-lanss' OR 'neuropathic pain symptom inventory' OR 'neuropathic pain questionnaire' OR 'id pain' OR 'neuropathic pain scale'

7	'magnetic resonance imaging' /exp OR 'diffusion tensor imaging'/exp OR 'ultrasonography'/exp
8	'prevalence'/exp OR 'incidence'/exp OR 'epidemiology'/exp
9	3 & 4
10	3 & 5
11	3 & 6
12	3 & 7
13	3, 4, & 8

Cinahl (EBSCO)

S1	Whiplash OR Whiplash injuries OR whiplash disorders OR motor vehicle collision
S2	MW cohort study OR case-control study OR observational study OR cross-sectional study
S3	(MW cohort study OR case-control study OR observational study OR cross-sectional study) AND (S1 AND S2)
S4	Peripheral nervous system disease OR somatosensory disorder OR neuropathic pain OR nerve disorder OR neuralgia OR peripheral nerve injury OR cervical plexus OR spinal nerve roots
S5	Neurological examination OR muscle strength OR electrodiagnosis OR neural conduction OR “dermatome” OR “myotome” OR “sympathetic reflexes” OR “small fibre neuropathy” OR “small fiber neuropathy” OR small fibre sensory neuropathy” OR “small fiber sensory neuropathy” OR “intraneural change” OR “quantitative sensory testing” OR muscle weakness OR sensory testing
S6	“Douleur Neuropathique 4 Questionnaire” OR “Leeds Assessment of Neuropathic Symptoms and Signs” OR “painDETECT” OR “DN4” OR “LANSS” OR S-LANSS” OR “Neuropathic Pain Symptom Inventory” OR “Neuropathic Pain Questionnaire” OR “ID Pain” OR “Neuropathic Pain Scale”
S7	Magnetic resonance imaging OR diffusion tensor imaging R ultrasonography
S8	Prevalence OR incidence OR epidemiology
S9	S3 AND S4
S10	S3 AND S5
S11	S3 AND S6
S12	S3 AND S7
S13	S3 AND S4 AND S8

Supplemental Table S2. Characteristics of included studies: design and population characteristics

Author & Date	Study Design	Study Participants	Female participants	Age of participants (years): mean (SD)
Sterling (2009)	Cross-sectional	WAD n=85	WAD n = 54	WAD=36.27 (12.69)
Smith (2013)	Cross-sectional	WAD n=90, controls n=30	WAD n=58, controls n=21	WAD recovered= 44.3 (10.4), Non-recovered=45.4 (9.7), controls=44.2 (9.7)
Karlsborg (1997)	Cohort	WAD n=34	WAD n=20	WAD=35.5
Henrikson (2013)	Cohort	WAD n=20, controls n=10	WAD n= 10	NA
Chuang (2002)	Cross-sectional	WAD n=85	NA	NA
Smith (2014)	Cohort	WAD n=53, controls n=30	WAD n=36, controls n=21	WAD=44.7 (10.9), controls=44.2 (9.7)
Sterling (2004)	Cohort	WAD n=80, controls n=20	WAD n=56, controls n=11	WAD=33.5 (14.7), controls= 39.5 (14.6)
Serrano-Munoz (2019)	Cohort	WAD n=20, controls n=15	WAD n=16, controls n=9	WAD No Pain (n=5)=40.8 (4.6), Pain (n=15)=39.7 (3.1), controls=40.5 (3.4)
Sterling (2010)	Cohort	WAD n=62, controls n=22	WAD n= 36, controls n= 14	WAD=36.1 (13.13), controls=40.1 (13.6)
Bowles (2004)	Cross-sectional	WAD n=25	WAD = 5	WAD = 37.5 (15.5)
Greening (2018)	Cross-sectional	WAD n= 9, controls n = 13	WAD n=6, controls n= 6	WAD =46.8 (2.9), controls=38.9 (10.8)
Hashish (2017)	Cross-sectional	WAD n=903	WAD n=488	WAD=34.3 (18.04)
Chien (2009)	Cross-sectional	WAD n=31, controls n=31	WAD n=25, controls n=25	WAD=35.3 (10.7), controls=31.4 (8.9)
Chien (2008b)	Cross-sectional	WAD n=52, controls n=31	WAD n=32, controls n=25	WAD=36.3 (13.1), controls=31.4 (8.9)
Vaegter (2018)	Cross-sectional	WAD n=108	WAD n=58	WAD=45.7 (11.6)
Greening (2005)	Cross-sectional	WAD n=9, controls n=8	WAD n=5, controls n=4	WAD=37.2 (9.87), controls=40.8 (8.2)
Pedler (2013)	Cross-sectional	WAD n=64, controls n=24	WAD n=35, controls n=14	WAD=44.7 (12.6), controls=40.3 (13.4)
Radanovf (1995)	Cohort	WAD n=117	WAD n=68	WAD =30.7 (9.6)
Alpar (2002)	Cohort	WAD n=38, controls n=30	WAD n=28, controls n=18	WAD=37.5, controls=34.2
Pettersson (1994)	Cohort	WAD n=39	WAD n=20	WAD=32 (range: 18-52)
Midha (1997)	Cross-sectional	WAD n=16	NA	NA

Miranda (2016)	Cross-sectional	WAD n=20	NA	NA
Jonsson (1994)	Cohort	WAD n=24	WAD n=13	WAD=36.5 (9.6)
Braddom (2009)	Case-control	WAD n=1,334	NA	WAD= 40
Kaiser (2014)	Cross-sectional	WAD n=12	NA	NA
Coert (1994)	Cross-sectional	WAD n=157	NA	NA
Sterling (2006)	Cohort	WAD n=65	WAD n=56	WAD=36.27 (12.69)
Sterling (2005)	Cohort	WAD n=76	WAD n=53	WAD=36.27 (12.69)
Sturzenegger (1994)	Cohort	WAD n=137	WAD n=81	WAD=30.8 (9.6)
Goudman (2020)	Case-control	WAD n=21, controls n=18	WAD n=10, controls n=11	WAD=45.8 (40.41–51.08), controls=46.8 (27.73 - 51.21)
Sterner (2001)	Cohort	WAD n=43	WAD n=24	WAD=36.2 (23–62)
Radanov (1994)	Cohort	WAD n=117	WAD n=68	WAD=30.8 (9.6)
Sterling (2002)	Cohort	WAD n=156, controls n=95	WAD n=127, controls n=50	WAD=37.43 (9.3), controls=38.95 (14.47)
Bekelis (2014)	Cohort	WAD n=384,539	WAD n=164,292	WAD=40.45 (19.33)
Lo (2007)	Cohort	WAD n=20	WAD n=10	WAD=42 (23–60)
Sterling (2003)	Cohort	WAD n=76, controls n=20	WAD n=54, controls n=12	WAD=36.27 (12.69), controls=40.1 (13.6)
Chien (2010)	Cross-sectional	WAD n=50, controls n=31	WAD n=39, controls n=25	WAD=37.2 (10.4), controls=31.4 (8.9)
Farrell (2020)	Cross-sectional	WAD n=24, controls n=24	WAD n=16	WAD=49 (15 IQR), controls=50 (17 IQR)
Squires (1996)	Cohort	WAD n=37	NA	WAD symptomatic=40.7, WAD asymptomatic=30.7
Chuang (1998)	Cross-sectional	WAD n=14	WAD n=6	WAD=38.26 (14.85)
Sturzenegger (1995)	Cohort	WAD n=117	WAD n=73	WAD=30.8 (9.5)
Saadat (2011)	Cross-sectional	WAD n=78	NA	NA
Moog (2002)	Case-control	WAD n=43, controls n=43	WAD n=28, controls n=28	WAD=37 (12-66), controls=NA
Sterling (2006)	Cohort	WAD n=76	WAD n=53	WAD=33.5 (14.7)
Wallin (2012)	Cohort	WAD n=28,	WAD n=28	WAD=40.1 (7.1)
Raak (2006)	Cohort	WAD n=17, controls n=18	WAD n=16, controls n=17	WAD=50.8 (11.3), controls=44.8 (10.2)
Mailis (1995)	Cohort	WAD n=32	WAD n=23	WAD=27.5 (23-55)
Kaiser (2012)	Cross-sectional	WAD n=75	WAD n=10	NA

Chien (2008a)	Cross-sectional	WAD n=50, controls n=31	WAD n=39, controls n=25	WAD=37.2 (10.4), controls=31.4 (8.9)
Maimaris (1988)	Cohort	WAD n=102	WAD n=58	WAD=37 (17-72)
Ovadia (2002)	Cohort	WAD n=866	WAD n=439	WAD=38 (26-50)
Steinberg (2005)	Cohort	WAD n=330	WAD n=193	WAD=19 (18)
Terzis (2009)	Cross-sectional	WAD n=25	NA	NA
Scott (2005)	Case-control	WAD n=29 controls n=20	WAD n=17 controls n=12	WAD=41.6 (10), controls=31.25 (10)

Supplemental Table S3. Quality Assessment using the Newcastle-Ottawa Scales

Author	Year	Selection	Comparability	Outcome	Total score
Cross-sectional		(/5)	(/2)	(/3)	(/10)
Sterling et al.	2009	4	0	3	7
Smith et al.	2013	4	2	2	8
Chuang et al.	2002	3	1	3	7
Bowles et al.	2004	2	0	3	5
Greening et al.	2018	4	2	3	9
Hashish et al.	2017	2	0	2	4
Chien A et al.	2009	4	1	2	7
Chien et al.	2008	4	1	2	7
Vaegter et al.	2018	5	0	2	7
Greening et al.	2005	4	1	3	8
Pedler et al.	2013	5	1	2	8
Midha	1997	3	0	2	5
Miranda et al.	2016	3	0	0	3
Kaiser et al.	2014	3	0	3	6
Coert et al.	1994	3	0	2	5
Chien et al.	2010	4	1	2	7
Farrell et al.	2020	5	2	3	10
Chuang et al.	1998	2	0	2	4
Saadat et al.	2011	2	1	2	5
Kaiser et al.	2012	3	0	3	6
Chien et al.	2008	4	1	3	8
Terziset al.	2009	3	0	0	3
Cohort		(/4)	(/2)	(/3)	(/9)
Karlsborg et al.	1997	2	0	3	5
Henrikson et al.	2013	0	1	3	4
Jull et al.	2014	3	2	3	8
Sterling et al.	2004	4	1	3	8
Serrano-Munoz	2019	3	1	2	6
Sterling	2010	3	1	3	7
Radanov et al.	1995	2	1	2	5
Alpar et al.	2002	2	0	3	5
Pettersson et al.	1994	2	0	3	5
Jonsson et al.	1994	2	0	3	5
Sterling et al.	2006	2	1	3	6
Sterling et al.	2005	2	1	3	6
Sturzenegger et al.	1994	3	1	1	5
Sterner et al.	2001	2	0	1	3
Radanov et al.	1994	3	1	2	6
Sterling et al.	2002	4	0	2	6
Bekelis et al.	2014	3	0	1	4
Lo et al.	2007	3	1	3	7
Sterling et al.	2003	2	1	3	6

Squires et al.	1996	1	0	2	3
Sturzenegger et al.	1995	3	1	2	6
Sterling et al.	2006	2	1	2	5
Wallin et al.	2012	1	1	2	4
Raak et al.	2006	1	0	2	3
Mailis et al.	1995	2	1	2	5
Maimaris et al.	1988	2	0	2	4
Ovadia et al.	2002	2	0	3	5
Steinberg et al.	2005	2	0	3	5
Case-control		(/4)	(/2)	(/3)	(/9)
Braddom et al.	2009	3	1	2	6
Goudman et al.	2020	3	1	1	5
Moog et al.	2002	4	1	1	6
Scott et al.	2005	3	1	1	5

Supplemental Table S4. Synthesis of results by type of outcome measure. Please note this does not include the sensory testing studies which are reported in Figure 2.

Measure	Number of studies	Number of participants	WAD grade	Outcome	Interpretation
S-LANSS[32,44,89,90,106]	4	208	I-III	A combined total n=71 WAD participants had positive scores ≥12. When subdivided by WAD II, 42 of 123 participants had scores ≥12.	34% of both the total WAD population as well as patients with WAD II had scores suggestive of neuropathic pain (≥12)
DN4[89]	1	20	I-III	No Pain (n=5): median 3 (IQR 1.5), Pain (n=15): median 4 (IQR 3) out of 10.	75% (of 20 participants) had scores suggestive of neuropathic pain (≥4).
NPSI[32,89]	2	44	I-III	WAD I-III: No Pain (n=5): median 0 (IQR 2), Pain (n=15): median 3 (IQR 6) out of 10.<br><br>WAD II: Mean (SD) score: 26.1 (18.3) out of 100.	Overall, participants had low severity (3/10 and 26.1/100) of neuropathic pain symptoms.
Neurological examination[2,32,44,55,58,62,66,73,76,79,80,92,107-110]	16	1,885	I-IV	n=241 pooled patients had positive findings	13% of pooled WAD patients had positive findings
Electrodiagnostic testing[2,11,12,19,20,22,50,56,57,62,67,68,73,83,94,115]	16	3,921	I-III	n=1,249 pooled participants had positive findings of peripheral nerve pathology	32% of pooled WAD participants had positive findings
Magnetic resonance imaging[44]	1	9	II	Greater T2 signal intensity (brachial plexus, median nerve - wrist): WAD mean= 0.52 ± 0.13 and 2.09 ± 0.33, respectively) compared to the control group (mean= 0.45 ± 0.07 and 1.38 ± 0.31, respectively; P<.05).	These findings suggest that patients with chronic whiplash may have peripheral nerve pathology measured at the wrist and brachial plexus.
Ultrasound[45]	1	9	II	WAD participants had significantly reduced longitudinal [mean=0.38 (0.08) mm, (95% CI=0.20–0.56 mm)] and transverse nerve movement [2.57 (0.80) mm, (95% CI=0.61–4.54 mm)] on the symptomatic side compared to the control group.	The results suggest nerve sliding impairments at both the forearm and the wrist compared to healthy controls.

Sympathetic vasoconstrictor reflex[101,102,104,105]	4	293	II-III	SVR results: Quotient of Integrals (QI) mean 59.42 (7.13). Sympathetic Reflex (SFR)= 0.72 (.70)	These findings may suggest sympathetic nerve dysfunction.
Laser evoked potentials[43]	1	21	I-III	No between-group differences (WAD and controls) for stimulus intensity (A-delta fibers), amplitudes and latencies of laser evoked potential wave components.	This study did not find a functional deficit in sensory afferent a-delta nerve fibers.
ICD-9 codes (nerve injury)[7,83]	2	384,617	NA	n=3,164 participants with ICD-9 codes for peripheral nerve injury	1% of pooled WAD data showed signs of nerve injury or neuropathic pain identified through ICD-9 codes.
Cutaneous silent periods[62]	1	20	I-III	18 of 20 participants demonstrated abnormal findings of at least one CSP recording (measured at hand and foot).	These findings may indicate peripheral nerve dysfunction, but larger studies are needed.
Intraepidermal nerve fiber density[32]	1	24	II	Index finger: WAD median (IQR) IENFD 4.5 (4.9) fibres/mm compared with 7.3 (3.9) fibres/mm for the control group (p = .010, r = .37.) Ankle: WAD mean [SD]: IENFD 7.3 [3.7] fibres/mm; control group: 9.3 [3.8] fibres/ mm (p = .09).	IENFD demonstrated structural peripheral nerve pathology at the index finger of participants with WAD II.
Nerve palpation[2,44,45,65]	4	88	I-III	An average of 91% (range 67-100%, n=56 total patients) and 94% (range 78-100%, n=50 total patients) had symptom reproduction with palpation of the median nerve and brachial plexus respectively.	Median nerve and brachial plexus palpation reproduced pain and symptoms suggestive of neural mechanosensitivity in a majority of patients in the included four studies.

Abbreviations: CSP: Cutaneous silent period; DN4: Douleur Neuropathique 4**;** ICD: International Classification of Diseases; IENFD: intraepidermal nerve fibre density; IQR: interquartile range; NPSI: Neuropathic Pain Symptom Inventory; NA: not available; SD: standard deviation; S-LANSS: Self-complete Leeds Assessment of Neuropathic Symptoms and Signs; WAD: whiplash associated disorders.
